# Impervious Surfaces Alter Soil Bacterial Communities in Urban Areas: A Case Study in Beijing, China

**DOI:** 10.3389/fmicb.2018.00226

**Published:** 2018-02-27

**Authors:** Yinhong Hu, Xiaolin Dou, Juanyong Li, Feng Li

**Affiliations:** ^1^State Key Laboratory of Urban and Regional Ecology, Research Center for Eco-Environmental Sciences, Chinese Academy of Sciences, Beijing, China; ^2^College of Resources and Environment, University of Chinese Academy of Sciences, Beijing, China; ^3^Key Laboratory of Coastal Environmental Processes and Ecological Remediation and Yantai Institute of Coastal Zone Research, Chinese Academy of Sciences, Yantai, China

**Keywords:** impervious surfaces, bacterial community, 16S rRNA gene sequencing, urbanization, land cover types

## Abstract

The rapid expansion of urbanization has caused land cover change, especially the increasing area of impervious surfaces. Such alterations have significant effects on the soil ecosystem by impeding the exchange of gasses, water, and materials between soil and the atmosphere. It is unclear whether impervious surfaces have any effects on soil bacterial diversity and community composition. In the present study, we conducted an investigation of bacterial communities across five typical land cover types, including impervious surfaces (concrete), permeable pavement (bricks with round holes), shrub coverage (*Buxus megistophylla Levl.*), lawns (*Festuca elata Keng ex E. Alexeev*), and roadside trees (*Sophora japonica Linn.*) in Beijing, to explore the response of bacteria to impervious surfaces. The soil bacterial communities were addressed by high-throughput sequencing of the bacterial 16S rRNA gene. We found that *Proteobacteria*, *Actinobacteria*, *Acidobacteria*, *Bacteroidetes*, *Chloroflexi*, and *Firmicutes* were the predominant phyla in urban soils. Soil from impervious surfaces presented a lower bacterial diversity, and differed greatly from other types of land cover. Soil bacterial diversity was predominantly affected by Zn, dissolved organic carbon (DOC), and soil moisture content (SMC). The composition of the bacterial community was similar under shrub coverage, roadside trees, and lawns, but different from beneath impervious surfaces and permeable pavement. Variance partitioning analysis showed that edaphic properties contributed to 12% of the bacterial community variation, heavy metal pollution explained 3.6% of the variation, and interaction between the two explained 33% of the variance. Together, our data indicate that impervious surfaces induced changes in bacterial community composition and decrease of bacterial diversity. Interactions between edaphic properties and heavy metals were here found to change the composition of the bacterial community and diversity across areas with different types of land cover, and soil properties play a more important role than heavy metals.

## Introduction

Urban populations and land areas have been increasing for decades, and land expansion rates are higher than or equal to population growth rates in many countries (Seto et al., 2011; Ezeh et al., 2012). Accompanying land urbanization, natural vegetation has been replaced with impervious surfaces, lawns, and greening trees, which have become the typical land cover types in urban areas (Deng and Wu, 2013; Wolch et al., 2014). It is estimated that nearly 580,000 km^2^ of the earth is covered by impervious surfaces, and this number is continuing to increase (Elvidge et al., 2007). China has more total area of impervious surfaces than any other country, with an increase of 53.3% from 2000 to 2008 (Kuang et al., 2013). Impervious surfaces impair urban ecosystem services by causing landscape fragmentation, habitat loss, and soil degradation (Van de Voorde et al., 2011; Seto et al., 2012). Previous studies have reported that impervious surfaces has significant effects on plants (Chen Y. et al., 2016; Chen et al., 2017), but the response of microorganisms to it remains unclear.

Soils are compacted when covered by impervious surfaces (Scalenghe and Marsan, 2009), and this compaction can alter bacterial communities for years afterward (Hartmann et al., 2012). Many studies have shown that installation of impervious surfaces decreased the microbial biomass, enzymatic activity, and functional diversity by impeding the exchange of gasses, water, and materials between soil and the atmosphere (Zhao et al., 2012; Wei et al., 2013; Piotrowska-Długosz and Charzyński, 2015). However, no studies have investigated soil microbial species diversity and community composition beneath impervious surfaces. With the recent development of high-throughput sequencing, Xu et al. (2014) found that urbanization did have an effect on the bacterial community by investigating urban park soils from cities with different levels of urbanization in China. In addition, Yan et al. (2016) investigated the soils of urban green space from different ring roads and found that urban development alters bacterial diversity and community composition. However, these studies overlooked impervious surfaces, even though they are the most common representatives of urbanization. Consequently, to fully understand the response of microbes to urbanization, it is necessary to learn about the effects of impervious surfaces on microbes through high-throughput sequencing. In view of the negative effects of impervious surfaces on the environment, ecologists have advocated using permeable pavement as an alternative to traditional impervious pavement (Brattebo and Booth, 2003; Scholz and Grabowiecki, 2007). Although many studies confirmed that permeable pavement systems could mitigate urban runoff (Fassman and Blackbourn, 2010; Kamali et al., 2017), the bacterial community activities under permeable pavement remain unclear.

Understanding the environmental factors influencing the microbial community is a fundamental goal in microbial ecology (Shen et al., 2015). Various studies have generally considered soil moisture to be the major factor influencing microbial community structure and enzyme activities (Brockett et al., 2012). Moreover, bacterial diversity and structure have been shown to be constrained by soil carbon and nitrogen (Chen C. et al., 2016; Francioli et al., 2016; Li et al., 2017). Soil carbon and nitrogen pools are depleted beneath impervious surfaces (Raciti et al., 2012; Yan et al., 2015). These altered the soil nitrogen transformation process and soil microbial activities in the study of Zhao et al. (2012). Heavy metals are one of the most widespread types of pollutant in urban areas (Wang et al., 2012). Zhao et al. (2013) reported that heavy metal content was the main factor influencing microbial biomass and microbial community functional diversity across land cover types in urban areas. In addition, plant species and temperature can influence bacterial communities (Jassey et al., 2013; Kai et al., 2016; Ridl et al., 2016). Ecologists can usually identify the controlling factor influencing the microbial community in forests (Shen et al., 2013), grasslands (Hu et al., 2014b; Kaiser et al., 2016) and farmlands (Zeng et al., 2016). Nevertheless, urban soils have been seriously affected by anthropogenic activity, and alteration of the bacterial community in urban areas is caused by many factors, and so cannot be easily predicted using common factors (Xu et al., 2014).

As described above, a better understanding of the effects of impervious surfaces on soil bacteria is essential to sustainable urban planning and improvement of ecological services of urban soil. Therefore, in this study, we quantified the soil bacterial relative abundance, diversity, and community composition across five typical land cover types, including impervious surfaces (concrete), permeable pavement (bricks with round holes), shrub coverage (*Buxus megistophylla Levl.*), lawns (*Festuca elata Keng ex E. Alexeev*), and roadside trees (*Sophora japonica Linn.*) in urban areas. Soil bacterial communities in different land cover types were investigated by high-throughput sequencing of the 16S rRNA gene. Specifically, we tested the hypotheses that soil bacterial community will be changed by land cover type, and that impervious surfaces will exert effects on bacterial communities. In order to test this hypothesis, we addressed the following questions. (1) How does impervious surfaces affect bacterial communities? (2) What are the primary factors influencing bacterial diversity and community composition across land cover types? and (3) Can permeable pavement improve microbial diversity and community composition?

## Materials and Methods

### Study Site and Soil Sampling

We selected the metropolitan city of Beijing, which is the capital of China with a population of over 20 million, as the study area (39°54′N, 116°24′E). This area has a temperate climate with a mean annual precipitation of 640 mm, a temperature of 12°C, and a predominant soil type of sandy loam based on the USDA texture classification system (United States Department of Agriculture, 2014). We collected samples from Beijing Olympic Park because it includes a variety of land cover types with a similar construction history (**Supplementary Figure S1**). The park was built for the 2008 Beijing Olympics. The previous land surface was 0.3–5.5 m deep backfilled layer, mainly composed of clayey silt, sandy silt and artificial deposits such as construction debris and waste products (Chen, 2013; Zhang et al., 2013, 2015). Soil used for backfilling was sourced from the nearby lake and foundation excavations (Zhang et al., 2015). Since the Beijing Olympic Games, the park has become an important tourist attraction as landmark of sports culture in China. We selected five typical land cover types (impervious surfaces, permeable pavement, shrub coverage, lawns, and roadside trees) to explore the response of bacteria to the impervious surfaces (**Supplementary Figure S1**). Soils under impervious surfaces came from roads that were about 2.5 m wide. Soils from permeable pavement were covered by bricks with round holes in the middle and on the edges, and covering about 100 square centimeters per brick. The shrub coverage sites and lawns sites were covered by *Buxus megistophylla Levl.* and *Festuca elata Keng ex E. Alexeev*, respectively. The roadside trees (*Sophora japonica Linn.*) were planted in a pit of approximately 1 square meters which was surrounded by impervious surfaces. Park rangers applied compound fertilizer and pesticides approximately one to three times per year to grasses, shrubs, and roadside trees.

Samples were collected in June 2016. The experimental design was a randomized complete block design with three randomly selected sites that were approximately 2 km apart. Each site was approximately 75 ha (500 m × 1500 m), including the five land cover types. At each site, three replicate plots (4 m × 4 m) were selected for each land cover type. In each subplot, six random soil samples (0–15 cm) were collected using a soil corer (2.5 cm diameter), then mixed thoroughly and pooled into one composite sample. Each sample was placed in a sterile plastic bag, sealed, and transported to the laboratory on ice. After removing the litter layer, roots, and stones, all samples were passed through a 2 mm sieve and then separated into three parts. One subsample was air-dried for analysis of physicochemical properties, one was stored at 4°C for microbial biomass determination, and the remainder was stored at -80°C for DNA extraction.

### Soil Geochemical Characteristics Analyses

Soil pH was determined with a soil to water ratio of 1:2.5 (w/v) using a pH meter (FE20-FiveEasy^TM^ pH, Mettler Toledo, Germany). The SMC was determined based on the weight of soils before and after being oven-dried for 48 h at 105°C. Soil total carbon (TC) and total nitrogen (TN) were determined using the Dumas method by an Element Analyser (Vario EL III, Elementar, Hanau, Germany). Soil organic carbon (SOC) content was determined by the dry combustion method with an Element Analyser (Vario EL III, Elementar, Hanau, Germany), using soil pretreated with HCl, and the soil organic matter content was 1.724 × SOC. The DOC was determined by UV adsorption at 254 nm (Brandstetter et al., 1996). Nitrate and ammonium were extracted with 2 mol l^-1^ KCl and quantified using a Continuous Flow Analyser (SAN++, Skalar, and Holand). Available potassium (AK) was extracted with NH_4_OAc and determined using ICP-OES (Bao, 2000). For soil heavy metal content analysis, samples were first digested using a four acid mixture containing 10 ml HCl, 5 ml HNO_3_, 5 ml HF, and 3 ml HClO_4_. The digested extracts were then diluted by ultrapure water to 50 ml for ICP-OES analysis of Cu and Zn, and for ICP-MS analysis of Cd, Cr, and Pb.

### DNA Extraction, Amplification, and High-Throughput Sequencing

Soil DNA was extracted from 0.5 g soil from each sample using the FastDNA^®^ SPIN kit for soil (MP Biomedicals, Santa Ana, CA, United States) according to the manufacturer’s instructions. The concentration and quality of the extracted DNA were assessed using a Nanodrop ND-1000 spectrophotometer (NanoDrop Technologies, Wilmington, DE, United States). The final soil DNA extracts were stored at -80°C until further analyzed. The V3–V4 regions of the 16S rRNA gene were subjected to high-throughput sequencing using the Illumina Miseq PE300 sequencing platform (Illumina, Inc., San Diego, CA, United States). The V3–V4 regions of bacterial 16S rRNA genes were sequenced and PCR amplified using the universal primers 336F (5′-GTACTCCTACGGGAGGCA GC A-3′) and 806R (5′-GTGGACTACHVGGGT WTCTAAT-3′) with incorporated sample barcode sequences. The PCR program was as follows: 95°C for 5 min, 25 cycles at 95°C for 30 s, 56°C for 30 s, and 72°C for 40 s with a final extension of 72°C for 10 min. The PCR products were separated by 1% agarose gel electrophoresis and the approximately 460 bp band was purified by using the Agencourt AMPure XP kit (Beckman Coulter, Inc., Brea, CA, United States). Sequencing was performed using the Illumina Miseq PE300 sequencing platform (Illumina, Inc., San Diego, CA, United States) according to the manufacturer’s recommendations. All sequences in this study are available in Sequence Read Achieve (SRA) database of NCBI under accession number SRP127237.

### Sequence Analysis

The extraction of high-quality sequences was performed with the Quantitative Insights into Microbial Ecology (QIIME) software package (version 1.4.0) (Caporaso et al., 2010). Reads not matching the primers or having read lengths below 300 were discarded. The quality-filtered reads were merged based on the overlap of the paired end read with the use of fastq-joint (Aronesty, 2011). The unique sequence set was clustered into operational taxonomic units (OTUs) (Blaxter et al., 2005) under the threshold of 97% identity using UCLUST. Chimeric sequences were identified and removed using USEARCH v.8 (Edgar, 2010). The taxonomy a representative sequence from each OTU was analyzed by UCLUST against the SILVA v. 119 16S rRNA database using a confidence threshold of 90%. After obtaining draft OTUs, singletons were removed to obtain the final quality results.

### Statistical Analysis

SPSS software v.18.0 (SPSS Inc., Chicago, IL, United States) and the vegan package of R v.3.1.1 (R Development Core Team, 2013) were used for statistical analysis. Conducted principal co-ordinates analysis (PCoA) on the basis of Bray-Curtis similarity distances was used to test the differences in microbial community composition across samples from different land cover types. Canonical correspondence analysis (CCA) was performed to show a visual relationship between environmental factors and bacterial distributions. CCA analysis was done with cca in R with a stepwise model from the vegan package. Mantel tests based on Bray–Curtis similarity distance was conducted to further identify the environmental factors that were significantly correlated to the community. The resulting clustering trees were paired with a heatmap of abundance data created with ‘heatmap.2’ from the ‘gplots’ package. Analysis of similarities (ANOSIM) was used to examine differences in bacterial community composition across land cover types. Furthermore, diversity (Chao 1 and Shannon index) was calculated in mothur (Schloss et al., 2009). In addition, analysis of variance (ANOVAs) were used for examining the differences in soil physicochemical properties, relative abundance of the main bacterial phyla, and diversity across land cover types (LSD; *P* = 0.05). The analysis of stepwise regression was used to verify relationships between bacterial diversity and environmental factors.

## Results

### Soil Characteristics

As shown in **Table 1**, there were significant differences in soil geochemical characteristics between impervious surfaces and other land cover types. All soil samples were alkaline, with pH values ranging from 8.3 ± 0.1 to 8.6 ± 0.2. The highest values were observed for impervious surfaces, and the lowest values were observed for roadside trees. The SMC of lawns (15.9%) was nearly 3.5 times that in permeable pavement (4.7%) and impervious surfaces (4.6%). The contents of TC, TN, SOC, DOC, NH_4_^+^-N, and AK under impervious surfaces were lower than that of other land cover types, but C:N ratio and NO_3_^-^-N showed the opposite trend. The heavy metal contents in different land cover types differed, but were all higher than the background content. The Pb, Cr, Cu, Zn, and Cd concentrations ranged from 31.0 ± 3.5 to 41.2 ± 4.4, 49.3 ± 4.7 to 68.2 ± 5.7, 40.6 ± 4.8 to 86.7 ± 7.9, 72.7 ± 8.3 to 140.5 ± 11.4, and 0.1 ± 0.0 to 0.4 ± 0.1 mg kg^-1^, respectively. The highest concentrations of Pb, Cr, Cu, and Zn were found in impervious surfaces, but the highest Cd was observed in permeable pavement. All metal concentrations were relatively low in roadside tree and shrub coverage areas.

**Table 1:** Soil geochemical characteristics across land cover types in Beijing, China.

Properties	Impervious surfaces	Permeable pavement	Shrub coverage	Lawns	Roadside trees
pH	8.6 ± 0.2a	8.3 ± 0.1c	8.4 ± 0.1b	8.4 ± 0.1b	8.3 ± 0.1c
SMC (%)	4.6 ± 1.0d	4.7 ± 0.8d	11.1 ± 2.2b	15.9 ± 2.9a	8.7 ± 1.4c
TC (g⋅kg^-1^)	12.1 ± 2.2b	17.4 ± 3.5a	19.5 ± 2.9a	18.9 ± 2.5a	19.4 ± 4.9a
SOC (g⋅kg^-1^)	2.2 ± 0.4e	8.9 ± 0.8b	11.9 ± 1.7a	5.6 ± 0.6d	6.7 ± 1.4c
DOC (mg⋅kg^-1^)	64.2 ± 8.8e	218.7 ± 2b	340.8 ± 25.6a	120.0 ± 12.5d	161.9 ± 16.1c
TN (mg⋅kg^-1^)	371.5 ± 36.9c	695.8 ± 120.4b	860.2 ± 122.7ab	760.7 ± 156.5ab	898.6 ± 307.4a
NH_4_^+^-N (mg⋅kg^-1^)	5.3 ± 0.9d	11.1 ± 1.2a	8.8 ± 0.8bc	9.5 ± 1.2b	8.4 ± 0.6c
NO_3_^-^-N (mg⋅kg^-1^)	6.6 ± 0.7a	5.4 ± 0.8b	3.2 ± 0.7c	3.5 ± 0.6c	3.9 ± 0.7c
C: N ratio	32.5 ± 4.3a	25.1 ± 3.4b	22.7 ± 1.6bc	25.3 ± 3.2b	21.7 ± 2.8c
AK (mg⋅kg^-1^)	113.4 ± 25.9d	289.2 ± 79.7ab	327.5 ± 56.9a	225.5 ± 35.0c	263.4 ± 101.3bc
Pb (mg⋅kg^-1^)	41.2 ± 4.4a	34.3 ± 3.3bc	31.0 ± 3.5d	32.4 ± 2.7cd	37.1 ± 6.0b
Cr (mg⋅kg^-1^)	68.2 ± 5.7a	53.5 ± 4.2cd	49.3 ± 4.7d	62.7 ± 4.3b	55.7 ± 1.9c
Cu (mg⋅kg^-1^)	86.7 ± 7.9a	53.6 ± 2.9b	40.6 ± 4.8d	46.8 ± 4.9c	43.9 ± 3.1cd
Zn (mg⋅kg^-1^)	140.5 ± 11.4a	79.8 ± 5.1b	74.7 ± 9.6b	78.8 ± 6.4b	72.7 ± 8.3b
Cd (mg⋅kg^-1^)	0.4 ± 0.1a	0.2 ± 0.0c	0.1 ± 0.0c	0.2 ± 0.1b	0.2 ± 0.1bc


**Data are expressed as mean ± SE, n = 9. Different letters indicate statistical significance at P < 0.05 among land cover types. SMC, soil moisture content; TC, total carbon; SOC, soil organic carbon; TN, total nitrogen; DOC, dissolved organic carbon; AK, available potassium*.*

### Taxonomic Distributions of Dominant Bacteria in Different Land Cover Types

To analyze the taxonomic compositions of the microbial community, a total of 1,987,125 16S rRNA V3-V4 sequences were generated from all 45 samples in this study, which varied from 23,065 to 62,506 sequences among samples (mean = 44,158) (**Supplementary Table S1**). High quality sequences were clustered into OTUs at the ≥ 97% similarity level, a process in which 9,172 unique OTUs were identified and individual samples contained 1,840–3,829 OTUs. All of the bacterial OTUs were classified into 42 different phyla, 104 classes, 249 orders, 493 families, and 975 genera.

**FIGURE 1:**
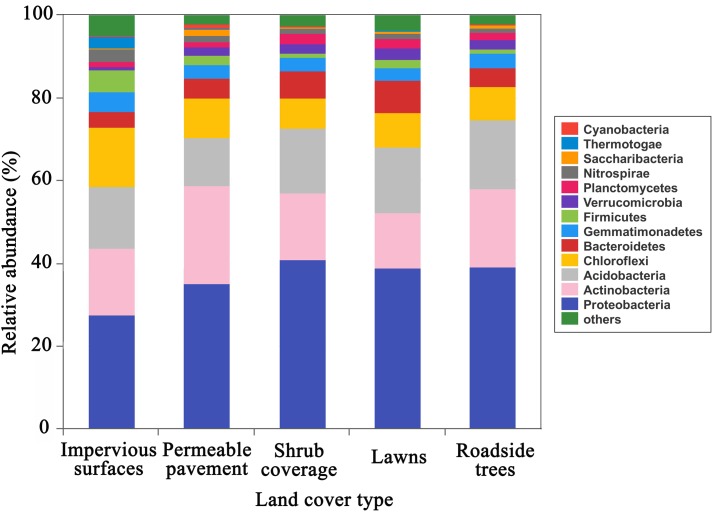
Relative abundance of the dominant bacterial community at the phylum level in samples separated by land cover type category. Relative abundances were found to depend on the average relative number of the bacterial sequences of nine samples from land cover type. Here “others” is given with respect to the taxa with a maximum abundance of < 0.5% in any sample.

The predominant phyla (relative abundance > 5%) included *Proteobacteria*, *Actinobacteria*, *Acidobacteria*, *Chloroflexi*, *Bacteroidetes*, and *Firmicutes*, together accounted for 82–89% of the total sequence data (**Figure 1**). *Proteobacteria, Actinobacteria, Acidobacteria*, and *Chloroflexi* were the predominant phyla in all soil samples. In particular, *Firmicutes* were the major phylum under impervious surfaces, while *Bacteroidetes* were the dominant phylum under shrub coverage and lawns. All of the major abundant phyla showed significant differences in relative abundance between the impervious surfaces and other land cover types (**Supplementary Table S2**). The highest level of *Proteobacteria* (40.9%), *Actinobacteria* (23.9%), *Acidobacteria* (16.5%), and *Bacteroidetes* (7.7%) appeared in shrub coverage, permeable pavement, roadside trees and lawns, respectively. *Chloroflexi* (14.4%) and *Firmicutes* (5.4%) were most prevalent beneath impervious surfaces.

As shown in **Supplementary Table S3**, there are 35 classes, 34 orders, 58 families, 35 genera, and 74 OTUs identified as dominant (relative abundance ≥ 0.5% in at least one sample), respectively. The most dominant classes in different land cover types were *Proteobacteria*, *Acidobacteria*, *Actinobacteria*, *Betaproteobacteria*, *Gammaproteobacteria*, *Deltaproteobacteria*, *Thermoleophilia*, and *Gemmatimonadetes.* The most dominant orders were *Rhizobiales*, *Subgroup_6*, and *Rhodospirillales*, which accounted for 13.7–24.3% of total bacteria in different land cover types. The most dominant families were *Nitrosomonadaceae*, *Anaerolineaceae*, and *Gemmatimonadaceae*, and their relative abundance was higher under impervious surfaces than beneath other types of land cover. At the genus level, the most abundant genera mainly included *Arthrobacter*, *Chryseolinea*, *Gaiella*, *Skermanella*, *Steroidobacter*, *Streptomyces*, and *Sphingomonas*. At the OTU level, the most abundant 74 OTUs accounted for 27.4% (impervious surfaces), 29.0% (lawns), 32.1% (permeable pavement), 34.2% (roadside trees), and 29.3% (shrub coverage) of total reads on average in different land cover types, respectively. These OTUs mainly belonged to the same abundant genera mentioned above.

### Bacterial Community Composition and Diversity

**FIGURE 2:**
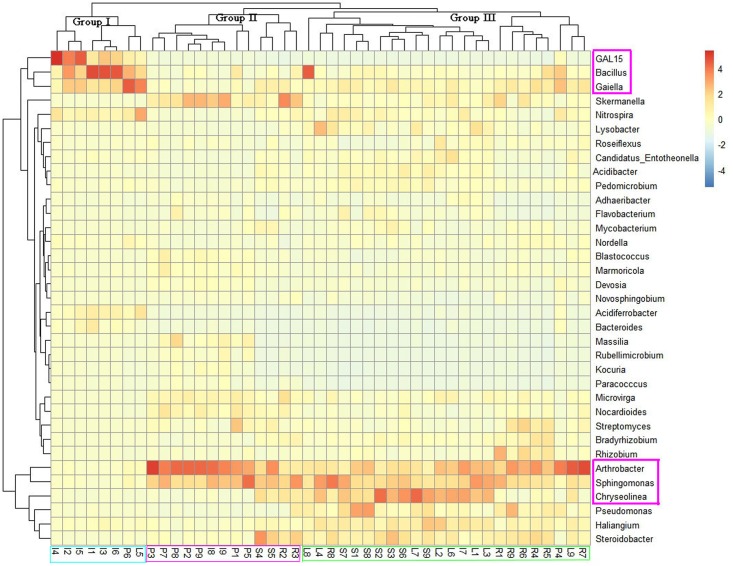
Heatmap of dominant genera of soil bacteria and cluster analysis of bacterial community composition across land cover types. The heatmap and clustering were computed from OTUs. The blue denotes low relative abundance across a bacterial taxon, and the red denotes high relative abundance.

To assess the microbial community structure of all samples, we conducted principal co-ordinates analysis (PCoA) on the basis of Bray–Curtis similarity distance at OTU level. As shown in **Supplementary Figure S2**, bacterial structures tended to be relatively similar among samples within the same land cover type and to differ distinctly across different types of land cover. The ANOSIM analysis revealed that the soil bacterial community differed significantly across land cover types at the OTU level (*R* = 0.49, *P* = 0.001). Three land cover types (shrub coverage, roadside trees and lawns) formed a cohesive group that was well separated from the other land cover types. Most of the impervious samples formed a cluster. While there were two impervious samples got together with permeable samples, but another two permeable samples were clustered with impervious samples. The analysis of microbial community heatmap and a multiple sample similarity tree were used to identify the similarity and differences of the four bacterial community structures. The bacterial communities of the 45 soil samples were roughly clustered into three groups (**Figure 2**). Group I consisted of six impervious samples, one permeable sample, and one lawn sample. Group II consisted of seven permeable samples, two roadside samples, two impervious samples, and two shrub samples. Group III contained samples from shrub coverage, lawns, and roadside trees. The relative abundance of *GAL15*, *Bacillus*, and *Gaiella* was higher in the most of the samples from impervious surfaces than in those of other land cover types, while the abundance of *Arthrobacter*, *Sphingomonas*, and *Chryseolinea* presented the opposite trend.

We conducted ANOSIM analysis to further assess the difference in bacterial community structure between impervious land cover and other types of land cover. There are more significant differences in bacterial community structure between impervious surfaces and shrub coverage (*R* = 0.46, *P* = 0.002), roadside trees (*R* = 0.45, *P* = 0.002), and lawns (*R* = 0.38, *P* = 0.005) than among communities associated with permeable pavement (*R* = 0.27, *P* = 0.03).

Rarefaction was performed to show the diversity of the samples. Both the Chao 1 index and Shannon index were used to compare the levels of bacterial diversity (**Table 2** and **Supplementary Figure S3**). Soil bacterial diversity was similar under shrub coverage, lawns, roadside trees, and permeable pavement. However, soils from impervious surfaces presented a lower bacterial diversity, and differed greatly from other land cover types. To verify the environmental factors that affected the bacterial diversity, correlation analysis between the environmental factors and Chao 1 and Shannon index was conducted through stepwise regression (**Table 3**). Correlation analysis showed that soil bacteria diversity was significantly correlated with Zn, DOC, and SMC.

**Table 2:** The alpha diversity of bacterial communities in different land cover types.

Land cover type	Shannon	Chao 1
Impervious surfaces	6.3 ± 0.3b	3315.2 ± 443.0c
Permeable pavement	6.7 ± 0.1a	4684.2 ± 328.3a
Shrub coverage	6.8 ± 0.1a	4590.6 ± 369.9a
Lawns	6.7 ± 0.1a	4205.0 ± 266.5b
Roadside trees	6.7 ± 0.1a	4675.4 ± 226.2a


**Data are expressed as mean ± SE, n = 9. Different letters indicate statistical significance at P < 0.05 among land cover types*.*

**Table 3:** Results of stepwise regression for the effects of soil properties on the alpha diversity of bacterial communities.

Index	Regression model	*R*^2^	*F*	*P*
Shannon	*y* = 6.646 – 0.390 (Zn) + 0.374 (DOC) + 0.257 (SMC)	0.616	24.497	0.000
Chao 1	*y* = 5299.579 – 14.381 (Zn) + 1.540 (DOC)	0.629	38.320	0.000


**The abbreviations for soil properties are the same as presented in **Table 1***.*

### Relationships between Environmental Parameters and Bacterial Community Composition

**FIGURE 3:**
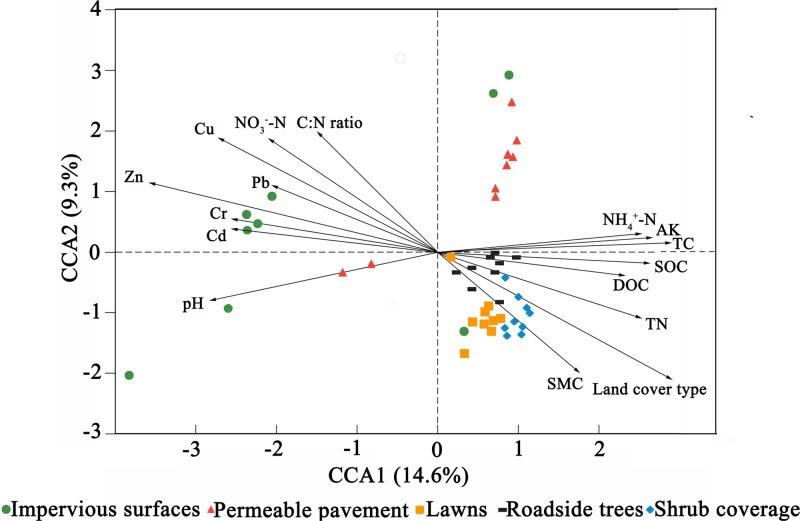
Canonical correspondence analysis (CCA) of the bacterial communities with symbols coded by land cover type.

Canonical correspondence analysis (CCA) was conducted to identify the environmental factors that could influence bacterial community variation among land cover types (**Figure 3**). The sixteen parameters (land cover type, pH, SMC, TC, TN, C:N ratio, SOC, DOC, NH_4_^+^-N, NO_3_^-^-N, AK, Pb, Cr, Cu, Zn, and Cd) were selected for analysis by CCA. The results indicated that Zn, NH_4_^+^-N, and land cover type could be the main drivers (longer arrow) influencing the bacterial community composition. Other factors such as Cu, C:N ratio, and NO_3_^-^-N also showed a high correlation with bacterial community composition based on a Mantel test (**Table 4**). Environmental variables that could influence bacterial community variation were different for different types of land cove (**Supplementary Figure S4**). The pH, Zn, and SMC could be the main drivers influencing the bacterial community composition beneath impervious surfaces, permeable pavement, and lawns, respectively. TC was the main factor shaping the composition of the bacterial community under shrub coverage and roadside trees.

**Table 4:** Mantel test results for the correlation between community composition and environmental variables for bacteria across land cover types.

Variable	*R*	*P*
Zn	0.60	0.001
Cu	0.59	0.001
NH_4_^+^-N	0.46	0.001
Land cover type	0.41	0.001
NO_3_^-^-N	0.39	0.001
C: N ratio	0.34	0.002
pH	0.32	0.003
AK	0.29	0.001
Cr	0.29	0.001
SMC	0.28	0.002
TN	0.27	0.001
Pb	0.27	0.004
SOC	0.25	0.001
TC	0.24	0.010
Cd	0.23	0.007
DOC	0.16	0.026


**The abbreviations for soil properties are the same as presented in **Table 1***.*

**FIGURE 4:**
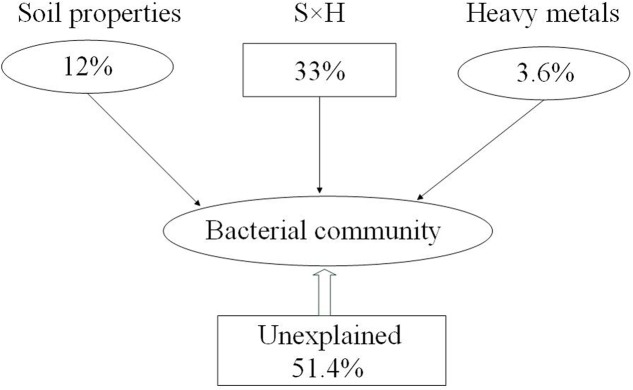
Variation partitioning analysis (VPA) of the effects of soil properties (S), heavy metals (H), and interactions between them on the bacterial community structure. Circles show the percentage of variation explained by each factor alone. The percentage of variation explained by interactions between the two factors is shown as squares. The unexplained variation is depicted in square on the bottom.

These results showed that both soil properties (NH_4_^+^-N, NO_3_^-^-N, TC, and C:N ratio) and pollutants (Cu and Zn) had effects on bacterial community composition. We divided those factors into two sections, including soil properties (pH, SMC, TC, TN, C:N ratio, SOC, DOC, NH_4_^+^-N, NO_3_^-^-N, AK) and heavy metals (Pb, Cr, Cu, Zn, and Cd) to calculate their relative contributions to the variance in the bacterial community. The relative contributions were assessed using variance partitioning analyses. As shown in **Figure 4**, 48.6% of the variance could be explained by these two groups of factors. Soil properties and heavy metals independently explain 12 and 3.6% of the total bacterial community variance, respectively. Interaction between edaphic properties and heavy metals explained 33% of the variance.

## Discussion

Modifications associated with urban land cover and its landscape pattern directly impact soil properties (Scharenbroch et al., 2005; Hu et al., 2007; Park et al., 2010). In most Chinese cities, the concentrations of heavy metals are higher than their background (Wei and Yang, 2010; Liu et al., 2016). In this work all soil samples were characterized as polluted in terms of Cd, Cu, Cr, Pb, and Zn accumulation (**Table 1**). This is closely related to the fact that the soil that was used for backfilling was composed of construction debris and waste products. Soils from construction wastes posed a risk in terms of Zn, Cu, Pb, Cr, Cd, and Ni contents (Ljung et al., 2006; Hu et al., 2014a; Gao et al., 2015). Moreover, the study area is located at the North 5th Ring Road of Beijing a road with heavy traffic load (≈300 000 vehicles day^-1^) (Qiao et al., 2011). Coal combustion and vehicle exhaust are important anthropogenic sources of heavy metals (Li et al., 2001; Xi et al., 2010; Luo et al., 2015). The heavy metal concentrations may also be affected by fertilizers and pesticides (Atafar et al., 2010; Alloway, 2013), and the use of compound fertilizer and pesticides may increase the level of soil heavy metals beneath lawns, shrubs, and roadside trees. Together these factors result in high accumulation levels of heavy metals across land cover types in urban areas. It is noteworthy that the soil from impervious surfaces suffered from the most severe contamination of heavy metals, particularly Zn and Cu, more than areas with other types of land cover. Soils containing construction waste show clearly higher concentrations of heavy metals than natural soil material (Abel et al., 2014). However, it is difficult to determine the contribution of each process (including soil backfill, construction processes, coal combustion and vehicle exhaust) to the concentrations of heavy metal. Many studies (Bieby et al., 2011; Ali et al., 2013) show that phytoremediation is an effective and affordable biological solution. It is used to extract or remove inactive metals and metal pollutants from contaminated soil. In the present study, the concentrations of heavy metals were relatively low under lawns, shrubs, and roadside trees, which indicates that the heavy metals may be continually removed by aboveground vegetation. In contrast, soil sealed by concrete could not release heavy metals to the rest of the world. Furthermore, soils carbon, nitrogen, and water were depleted beneath impervious surfaces because they are completely separated from the atmosphere (Lorenz and Lal, 2009). The magnitude of the loss of soil carbon and nitrogen beneath impervious surfaces is unknown, but likely possibilities include loss to the atmosphere as CO_2_, N_2_O, and N_2_; or aqueous losses as dissolved organic and inorganic carbon and nitrogen (Raciti et al., 2012).

Our results demonstrated that different types of land cover altered the bacterial community composition (**Supplementary Figure S2**). We found that *Proteobacteria*, *Actinobacteria*, *Acidobacteria*, *Chloroflexi*, *Bacteroidetes*, and *Firmicutes* were the most abundant phyla and were ubiquitous in urban soils. The predominance of the phyla *Proteobacteria*, *Firmicutes*, and *Chloroflexi* under in soil of impervious surfaces differed significantly with other types of land cover (**Supplementary Table S2**). *Proteobacteria* has been reported to be positively related to soil nutrient availability (e.g., TC and TN) (Koyama et al., 2014; Delgado-Baquerizo et al., 2016), and they less abundant under impervious surfaces than other types of land cover. While higher amounts of *Firmicutes* and *Chloroflexi* in soil of impervious surfaces indicated that *Firmicutes* and *Chloroflexi* were favored under impervious conditions. This may be because they were resistant to drought and extreme environmental changes (Battistuzzi and Hedges, 2009; Mendes et al., 2015). Soil bacterial community composition beneath impervious surfaces differed across the remaining types of land cover (PCoA, CCA, ANOSIM, and heatmap analyses). The difference in soil bacterial community composition between impervious surfaces and permeable pavement was small compared with other land cover types (*R* = 0.27, *P* = 0.03).

Soil microbial diversity was found to be sensitive to perturbations because of anthropogenic activities and pollution (Torsvik et al., 1998; Moffett et al., 2003; Fei et al., 2010). Xu et al. (2014) suggested that soil bacterial diversity did not differ in green spaces among cities in China, but Yan et al. (2016) reported it differed significantly across urban ring road areas. In the present work, soil bacterial diversity under impervious surfaces was low and differed dramatically from other land cover types, but there were no differences among the remaining four types of land cover, indicating that impervious surfaces decreased soil bacterial diversity (**Table 2**). In contrast, permeable pavement maintained a similar level of soil bacterial diversity with shrub coverage, roadside trees, and lawns. These results were similar to those of a previous study that showed that semi-impervious surfaces systems could reduce the negative effects of impervious surfaces in urban paved areas (Piotrowska-Długosz and Charzyński, 2015). It should be noted that this study examined only one type of permeable pavements; therefore, more types should be considered in future studies. Furthermore, soils show clear spatial heterogeneity in urban areas, and multiple factors affect soil microbial diversity. For example, water stress modifies the microbial community structure (Hueso et al., 2012), and changes in availability of substrates may result in predictable shifts in soil bacterial diversity (Goldfarb et al., 2011). Heavy metal pollution decreases microbial diversity and activity (Chen et al., 2014; Xie et al., 2016). Here, we found that bacterial diversity was mainly correlated with Zn, DOC, and SMC across land cover types (**Table 3**). These findings indicate that these three factors had important effects on affecting bacterial distribution patterns in different land cover types.

Environmental variables interact, change, and play key roles in shaping bacterial community composition in many ecosystems (Drenovsky et al., 2004; Fierer and Jackson, 2006; Berg and Smalla, 2009). In the present study, stepwise regression (**Table 3**) and Mantel test (**Table 4**) showed that Zn was closely correlated with bacterial diversity and community composition across land cover types. While different environmental factors influenced the variation in bacterial communities in different land cover types (**Supplementary Figure S4**). Zn was the main factor shaping the bacterial community composition under permeable pavement. Soil properties, such as pH, SMC, and TC, were the chief factors in the remaining types of land cover. Although, some studies have demonstrated that Zn is often correlated with bacterial community composition (Wang et al., 2007; Gołȩbiewski et al., 2014; Xu et al., 2017), we did not ensure Zn to be the most predominant factor affecting bacterial communities across land cover types. Because urban soils have been seriously affected by anthropogenic activities (Chen et al., 2005; Karim et al., 2014), and alterations in the bacterial community in urban areas are caused by many factors, community structure cannot be easily predicted by one factor (Xu et al., 2014). The causal connection between them needs more experiments to confirm. Besides Zn, Cu, NH_4_^+^-N, NO_3_^-^-N, and C:N ratio also correlated with bacterial community composition (**Figure 3** and **Table 4**). Zhao et al. (2013) had reported that nutrient availability and heavy metals content were the key factors influencing microbial biomass and functional diversity in urban soils. Here, we divided the environmental factors into two groups based on soil properties and pollution factors to determine which are more important in shaping the composition of the bacterial community. Based on VPA results (**Figure 4**), 48.6% of the bacterial community variance could be explained by these two groups. It is reasonable to expect that some additional factors, such as soil temperature, soil texture, water-retention capacity, and other chemicals, play key roles in shaping bacterial community composition in urban soil. Interactions between edaphic properties and heavy metals contributed to 33% of the community variance. This meant soil bacterial community variance is caused by changes in soil properties and heavy metal content. Soil properties were here found to be more important than heavy metals in determining the distribution of bacterial communities across land cover types.

## Conclusion

In summary, the present study showed that the major bacterial phyla across land cover types were *Proteobacteria, Actinobacteria, Acidobacteria, Bacteroidetes, Chloroflexi, Gemmatimonadetes*, and *Firmicutes* in urban areas. Impervious surfaces altered bacterial community composition, and decreased bacterial diversity. Soil bacterial diversity was mainly correlated with Zn, DOC, and SMC. Interaction between edaphic properties and heavy metals changed bacterial community composition across land cover types. Soil properties, such as NH_4_^+^-N, NO_3_^-^-N, C:N ratio, and TC, played a more important role than heavy metal pollution in shaping urban soil bacterial community composition. Although permeable pavement is beneficial to the overall diversity of bacterial communities compared to impervious surfaces, we examined only a single type of permeable pavement. More types of permeable pavement should be considered in the future to study their effects on microbial communities. Moreover, we focused only on bacteria, and were not concerned with fungi and other microorganisms. For a better understanding of the biogeochemical cycling of soil carbon and nitrogen beneath impervious surfaces and to provide a reference for urban land planning, further studies on microbial ecology in urban areas should focus on fungus and functional microbes. Such information will be helpful for ecological restoration and management of soil in urban environments.

## Author Contributions

FL designed the study. YH designed the study, determined the soil physicochemical properties, analyzed the sequencing data, and wrote the manuscript. JL collected soil samples and determined the soil physicochemical properties. XD helped revise the manuscript.

## Conflict of Interest Statement

The authors declare that the research was conducted in the absence of any commercial or financial relationships that could be construed as a potential conflict of interest.

## Supplementary Material

**FIGURE S1 |** Sampling sites in the urban area of Beijing (created using ArcGIS 10.1), and the pictures of different land cover types.

**FIGURE S2 |** Principal coordinates analysis (PCoA) of Bray–Curtis similarity distance of the bacterial communities comparing all 45 samples from the five land cover types. Sites have been color-coded according to land cover type.

**FIGURE S3 |** Rarefaction curves for Chao 1 index **(A)** and Shannon index **(B)** at the level of 97% 16S rRNA gene similarity across land cover types.

**FIGURE S4 |** Canonical correspondence analysis (CCA) of the bacterial communities of each land cover type.

**TABLE S1 |** Number of sequence reads [before (BF) and after (AF) quality filtering using the Quantitative Insights into Microbial Ecology (QIIME) software package] and OTUs classified from after quality filtering sequences. Different letters indicate different land cover types. I, Impervious surfaces; L, Lawns; P, Permeable pavement; R, Roadside trees; S, Shrub coverage.

**TABLE S2 |** Relative abundance of the main bacterial phyla classified with RDP taxonomy in five land cover types (values represent % of total non-redundant sequences). Different letters indicate statistical significance at *P* < 0.05 among land cover types.

**TABLE S3 |** Relative abundances of the dominant bacterial classes **(a)**, orders **(b)**, families **(c)**, genera **(d)** and OTUs **(e)** across land cover types, and only those with relative abundance ≥0.5% at least at one sampling time were shown.

## References

[B1] Abel S. Nehls T. Mekiffer B. Wessolek G. (2014). Heavy metals and benzo[a]pyrene in soils from construction and demolition rubble. *J. Soils Sediments* 15 1–10. 10.1007/s11368-014-0959-4

[B2] Ali H. Khan E. Sajad M. A. (2013). Phytoremediation of heavy metals-concepts and applications. *Chemosphere* 91 869–881. 10.1016/j.chemosphere.2013.01.075 23466085

[B3] Alloway B. J. (2013). *Sources of Heavy Metals and Metalloids in Soils: Heavy Metals in Soils.* New York, NY: Springer, 10–50. 10.1007/978-94-007-4470-7_2

[B4] Aronesty E. (2011). *Command-Line Tools for Processing Biological Sequencing Data.* Durham, NC: Ea-utils.

[B5] Atafar Z. Mesdaghinia A. Nouri J. Homaee M. Yunesian M. Ahmadimoghaddam M. (2010). Effect of fertilizer application on soil heavy metal concentration. *Environ. Monit. Assess.* 160 83–89. 10.1007/s10661-008-0659-x 19058018

[B6] Bao S. D. (2000). *Soil Agricultural Chemical Elements Analysis.* Beijing: China Agriculture Press.

[B7] Battistuzzi F. U. Hedges S. B. (2009). A major clade of prokaryotes with ancient adaptations to life on land. *Mol. Biol. Evol.* 26 335–343. 10.1093/molbev/msn247 18988685

[B8] Berg G. Smalla K. (2009). Plant species and soil type cooperatively shape the structure and function of microbial communities in the rhizosphere. *FEMS Microbiol. Ecol.* 68 1–13. 10.1111/j.1574-6941.2009.00654.x 19243436

[B9] Bieby V. T. Siti R. S. A. Hassan B. Mushrifah I. Nurina A. Muhammad M. (2011). A review on heavy metals (As, Pb, and Hg) uptake by plants through phytoremediation. *Int. J. Chem. Eng.* 8 1687X–1806X. 10.1155/2011/939161

[B10] Blaxter M. Mann J. Chapman T. Thomas F. Whitton C. Floyd R. (2005). Defining operational taxonomic units using DNA barcode data. *Philos. Trans. Biol. Sci.* 360 1935–1943. 10.1098/rstb.2005.1725 16214751 PMC1609233

[B11] Brandstetter A. Sletten R. S. Mentler A. Wenzel W. W. (1996). Estimating dissolved organic carbon in natural waters by uv absorbance (254 nm). *J. Plant Nutr. Soil Sci.* 159 605–607. 10.1002/jpln.1996.3581590612

[B12] Brattebo B. O. Booth D. B. (2003). Long-term stormwater quantity and quality performance of permeable pavement systems. *Water Res.* 37 4369–4376. 10.1016/S0043-1354(03)00410-X 14511707

[B13] Brockett B. F. Prescott C. E. Grayston S. J. (2012). Soil moisture is the major factor influencing microbial community structure and enzyme activities across seven biogeoclimatic zones in western Canada. *Soil Biol. Biochem.* 44 9–20. 10.1016/j.soilbio.2011.09.003

[B14] Caporaso J. G. Kuczynski J. Stombaugh J. Bittinger K. Bushman F. D. Costello E. K. (2010). QIIME allows analysis of high-throughput community sequencing data. *Nat. Methods* 7 335–336. 10.1038/nmeth.f.303 20383131 PMC3156573

[B15] Chen C. Zhang J. Lu M. Qin C. Chen Y. Yang L. (2016). Microbial communities of an arable soil treated for 8 years with organic and inorganic fertilizers. *Biol. Fertil. Soils* 52 455–467. 10.1007/s00374-016-1089-5

[B16] Chen J. He F. Zhang X. Sun X. Zheng J. Zheng J. (2014). Heavy metal pollution decreases microbial abundance, diversity and activity within particle-size fractions of a paddy soil. *FEMS Microbiol. Ecol.* 87 164–181. 10.1111/1574-6941.12212 24020402

[B17] Chen T. B. Zheng Y. M. Lei M. Huang Z. C. Wu H. T. Chen H. (2005). Assessment of heavy metal pollution in surface soils of urban parks in Beijing, china. *Chemosphere* 60 542–551. 10.1016/j.chemosphere.2004.12.072 15950046

[B18] Chen W. (2013). *Carbon and Water Fluxes of Urban Green-land Ecosystem-case Study of Beijing Olympic Forest Park.* Doctoral dissertation, Beijing Forestry University, Beijing.

[B19] Chen Y. Wang X. Jiang B. Wen Z. Yang N. Li L. (2017). Tree survival and growth are impacted by increased surfaces temperature on paved land. *Landsc. Urban Plan.* 162 68–79. 10.1016/j.landurbplan.2017.02.001

[B20] Chen Y. Wang X. Jiang B. Yang N. Li L. (2016). Pavement induced soil warming accelerates leaf budburst of ash trees. *Urban For. Urban Green.* 16 36–42. 10.1016/j.ufug.2016.01.014

[B21] Delgado-Baquerizo M. Maestre F. T. Reich P. B. Trivedi P. Osanai Y. Liu K. (2016). Carbon content and climate variability drive global soil bacterial diversity patterns. *Ecol. Monogr.* 86 373–390. 10.1002/ecm.1216

[B22] Deng C. Wu C. (2013). A spatially adaptive spectral mixture analysis for mapping subpixel urban impervious surfaces distribution. *Remote Sens. Environ.* 133 62–70. 10.1016/j.rse.2013.02.005

[B23] Drenovsky R. E. Vo D. Graham K. J. Scow K. M. (2004). Soil water content and organic carbon availability are major determinants of soil microbial community composition. *Microb. Ecol.* 48 424–430. 10.1007/s00248-003-1063-2 15692862

[B24] Edgar R. C. (2010). Search and clustering orders of magnitude faster than BLAST. *Bioinformatics* 26 2460–2461. 10.1093/bioinformatics/btq461 20709691

[B25] Elvidge C. D. Tuttle B. T. Sutton P. C. Baugh K. E. Howard A. T. Milesi C. (2007). Global distribution and density of constructed impervious surfaces. *Sensors* 7 1962–1979. 10.3390/s7091962 28903208 PMC3841857

[B26] Ezeh A. C. Bongaarts J. Mberu B. (2012). Global population trends and policy options. *Lancet* 380 142–148. 10.1016/S0140-6736(12)60696-5 22784532

[B27] Fassman E. A. Blackbourn S. (2010). Urban runoff mitigation by a permeable pavement system over impermeable soils. *J. Hydrol. Eng.* 15 475–485. 10.1061/(ASCE)HE.1943-5584.0000238

[B28] Fei W. Yao J. Yang S. Chen H. Russel M. Chen K. (2010). Short-time effect of heavy metals upon microbial community activity. *J. Hazard. Mater.* 173 510–516. 10.1016/j.jhazmat.2009.08.114 19748181

[B29] Fierer N. Jackson R. B. (2006). The diversity and biogeography of soil bacterial communities. *Proc. Natl. Acad. Sci. U.S.A.* 103 626–631. 10.1073/pnas.0507535103 16407148 PMC1334650

[B30] Francioli D. Schulz E. Lentendu G. Wubet T. Buscot F. Reitz T. (2016). Mineral vs. organic amendments: microbial community structure, activity and abundance of agriculturally relevant microbes are driven by long-term fertilization strategies. *Front. Microbiol.* 7:1446. 10.3389/fmicb.2016.01446 27683576 PMC5022044

[B31] Gao X. Gu Y. Xie T. Zhen G. Huang S. Zhao Y. (2015). Characterization and environmental risk assessment of heavy metals in construction and demolition wastes from five sources (chemical, metallurgical and light industries, and residential and recycled aggregates). *Environ. Sci. Pollut. Res. Int.* 22 9332–9344. 10.1007/s11356-014-4058-2 25601613

[B32] Goldfarb K. C. Karaoz U. Hanson C. A. Santee C. A. Bradford M. A. Treseder K. K. (2011). Differential growth responses of soil bacterial taxa to carbon substrates of varying chemical recalcitrance. *Front. Microbiol.* 2:94. 10.3389/fmicb.2011.00094 21833332 PMC3153052

[B33] Gołȩbiewski M. Deja-Sikora E. Cichosz M. Tretyn A. Wróbel B. (2014). 16S rDNA pyrosequencing analysis of bacterial community in heavy metals polluted soils. *Microb. Ecol.* 67 635–647. 10.1007/s00248-013-0344-7 24402360 PMC3962847

[B34] Hartmann M. Howes C. G. Vaninsberghe D. Yu H. Bachar D. Christen R. (2012). Significant and persistent impact of timber harvesting on soil microbial communities in northern coniferous forests. *ISME J.* 6 2199–2218. 10.1038/ismej.2012.84 22855212 PMC3504969

[B35] Hu K. L. Li H. Li B. G. Huang Y. F. (2007). Spatial and temporal patterns of soil organic matter in the urban-rural transition zone of Beijing. *Geoderma* 141 302–310. 10.1016/j.geoderma.2007.06.010

[B36] Hu Y. Wang D. Wei L. Song B. (2014a). Heavy metal contamination of urban topsoils in a typical region of Loess Plateau, China. *J. Soils Sediments* 14 928–935. 10.1007/s11368-013-0820-1

[B37] Hu Y. Xiang D. Veresoglou S. D. Chen F. Chen Y. Hao Z. (2014b). Soil organic carbon and soil structure are driving microbial abundance and community composition across the arid and semi-arid grasslands in northern China. *Soil Biol. Biochem.* 77 51–57. 10.1016/j.soilbio.2014.06.014

[B38] Hueso S. García C. Hernández T. (2012). Severe drought conditions modify the microbial community structure, size and activity in amended and unamended soils. *Soil Biol. Biochem.* 50 167–173. 10.1016/j.soilbio.2012.03.026

[B39] Jassey V. E. Chiapusio G. Binet P. Buttler A. Laggoun-Défarge F. Delarue F. (2013). Above-and belowground linkages in *Sphagnum* peatland: climate warming affects plant-microbial interactions. *Glob. Change Biol.* 19 811–823. 10.1111/gcb.12075 23504838

[B40] Kai M. Effmert U. Piechulla B. (2016). Bacterial-plant-interactions: approaches to unravel the biological function of bacterial volatiles in the rhizosphere. *Front. Microbiol.* 7:108. 10.3389/fmicb.2016.00108 26903987 PMC4746483

[B41] Kaiser K. Wemheuer B. Korolkow V. Wemheuer F. Nacke H. Schöning I. (2016). Driving forces of soil bacterial community structure, diversity, and function in temperate grasslands and forests. *Sci. Rep.* 6:33696. 10.1038/srep33696 27650273 PMC5030646

[B42] Kamali M. Delkash M. Tajrishy M. (2017). Evaluation of permeable pavement responses to urban surface runoff. *J. Environ. Manage.* 187 43–53. 10.1016/j.jenvman.2016.11.027 27875770

[B43] Karim Z. Qureshi B. A. Mumtaz M. Qureshi S. (2014). Heavy metal content in urban soils as an indicator of anthropogenic and natural influences on landscape of Karachi - A multivariate spatio-temporal analysis. *Ecol. Indic.* 42 20–31. 10.1016/j.ecolind.2013.07.020

[B44] Koyama A. Wallenstein M. D. Simpson R. T. Moore J. C. (2014). Soil bacterial community composition altered by increased nutrient availability in arctic tundra soils. *Front. Microbiol.* 5:516. 10.3389/fmicb.2014.00516 25324836 PMC4183186

[B45] Kuang W. Liu J. Zhang Z. Lu D. Xiang B. (2013). Spatiotemporal dynamics of impervious surfaces areas across China during the early 21st century. *Chin. Sci. Bull.* 58 1691–1701. 10.1007/s11434-012-5568-2

[B46] Li F. Chen L. Zhang J. Yin J. Huang S. (2017). Bacterial community structure after long-term organic and inorganic fertilization reveals important associations between soil nutrients and specific taxa involved in nutrient transformations. *Front. Microbiol.* 8:187. 10.3389/fmicb.2017.00187 28232824 PMC5298992

[B47] Li X. Poon C. S. Liu P. S. (2001). Heavy metal contamination of urban soils and street dusts in Hong Kong. *Appl. Geochem.* 16 1361–1368. 10.1016/S0883-2927(01)00045-2

[B48] Liu R. Wang M. Chen W. Peng C. (2016). Spatial pattern of heavy metals accumulation risk in urban soils of Beijing and its influencing factors. *Environ. Pollut.* 210 174–181. 10.1016/j.envpol.2015.11.044 26716731

[B49] Ljung K. Otabbong E. Selinus O. (2006). Natural and anthropogenic metal inputs to soils in urban Uppsala, Sweden. *Environ. Geochem. Health* 28 353–364. 10.1007/s10653-005-9031-6 16724242

[B50] Lorenz K. Lal R. (2009). Biogeochemical C and N cycles in urban soils. *Environ. Int.* 35 1–8. 10.1016/j.envint.2008.05.006 18597848

[B51] Luo X. S. Xue Y. Wang Y. L. Cang L. Xu B. Ding J. (2015). Source identification and apportionment of heavy metals in urban soil profiles. *Chemosphere* 127 152–157. 10.1016/j.chemosphere.2015.01.048 25698100

[B52] Mendes L. W. Brossi M. J. L. Kuramae E. E. Tsai S. M. (2015). Land-use system shapes soil bacterial communities in southeastern Amazon region. *Appl. Soil Ecol.* 95 151–160. 10.1016/j.apsoil.2015.06.005

[B53] Moffett B. F. Nicholson F. A. Uwakwe N. C. Chambers B. J. Harris J. A. Hill T. C. J. (2003). Zinc contamination decreases the bacterial diversity of agricultural soil. *FEMS Microbiol. Ecol.* 43 13–19. 10.1111/j.1574-6941.2003.tb01041.x 19719692

[B54] Park S. J. Cheng Z. Yang H. Morris E. E. Sutherland M. McSpadden Gardener B. B. (2010). Differences in soil chemical properties with distance to roads and age of development in urban areas. *Urban Ecosyst.* 13 483–497. 10.1007/s11252-010-0130-y

[B55] Piotrowska-Długosz A. Charzyński P. (2015). The impact of the soil sealing degree on microbial biomass, enzymatic activity, and physicochemical properties in the Ekranic Technosols of Toruń (Poland). *J. Soils Sediments* 15 47–59. 10.1007/s11368-014-0963-8

[B56] Qiao Q. Zhang C. Huang B. Piper J. D. A. (2011). Evaluating the environmental quality impact of the 2008 Beijing Olympic Games: magnetic monitoring of street dust in Beijing Olympic Park. *Geophys. J. Int.* 187 1222–1236. 10.1111/j.1365-246X.2011.05195.x

[B57] R Development Core Team (2013). *R: A Language and Environment for Statistical Computing.* Vienna: R Foundation for Statistical Computing.

[B58] Raciti S. M. Hutyra L. R. Finzi A. C. (2012). Depleted soil carbon and nitrogen pools beneath impervious surfaces. *Environ. Pollut.* 164 248–251. 10.1016/j.envpol.2012.01.046 22377903

[B59] Ridl J. Kolar M. Strejcek M. Strnad H. Stursa P. Paces J. (2016). Plants rather than mineral fertilization shape microbial community structure and functional potential in legacy contaminated soil. *Front. Microbiol.* 7:995. 10.3389/fmicb.2016.00995 27446035 PMC4919359

[B60] Scalenghe R. Marsan F. A. (2009). The anthropogenic sealing of soils in urban areas. *Landsc. Urban Plan.* 90 1–10. 10.1007/s11356-016-8209-5 27987118 PMC5350235

[B61] Scharenbroch B. C. Lloyd J. E. Johnson-Maynard J. L. (2005). Distinguishing urban soils with physical, chemical, and biological properties. *Pedobiologia* 49 283–296. 10.1016/j.pedobi.2004.12.002

[B62] Schloss P. D. Westcott S. L. Ryabin T. Hall J. R. Hartmann M. Hollister E. B. (2009). Introducing mothur: open-source, platform-independent, community-supported software for describing and comparing microbial communities. *Appl. Environ. Microbiol.* 75 7537–7541. 10.1128/AEM.01541-09 19801464 PMC2786419

[B63] Scholz M. Grabowiecki P. (2007). Review of permeable pavement systems. *Build. Environ.* 42 3830–3836. 10.1016/j.buildenv.2006.11.016

[B64] Seto K. C. Fragkias M. Güneralp B. Reilly M. K. (2011). A meta-analysis of global urban land expansion. *PLOS ONE* 6:e23777. 10.1371/journal.pone.0023777 21876770 PMC3158103

[B65] Seto K. C. Güneralp B. Hutyra L. R. (2012). Global forecasts of urban expansion to 2030 and direct impacts on biodiversity and carbon pools. *Proc. Natl. Acad. Sci. U.S.A.* 109 16083–16088. 10.1073/pnas.1211658109 22988086 PMC3479537

[B66] Shen C. Ni Y. Liang W. Wang J. Chu H. (2015). Distinct soil bacterial communities along a small-scale elevational gradient in alpine tundra. *Front. Microbiol.* 6:582. 10.3389/fmicb.2015.00582 26217308 PMC4493907

[B67] Shen C. Xiong J. Zhang H. Feng Y. Lin X. Li X. (2013). Soil pH drives the spatial distribution of bacterial communities along elevation on Changbai Mountain. *Soil Biol. Biochem.* 57 204–211. 10.1016/j.soilbio.2012.07.013

[B68] Torsvik V. Daae F. L. Sandaa R.-A. Øvreås L. (1998). Novel techniques for analysing microbial diversity in natural and perturbed environments. *J. Biotechnol.* 64 53–62. 10.1016/S0168-1656(98)00103-5 9823658

[B69] United States Department of Agriculture (2014). *Keys to Soil Taxonomy.* Washington, DC: United States Department of Agriculture.

[B70] Van de Voorde T. Jacquet W. Canters F. (2011). Mapping form and function in urban areas: an approach based on urban metrics and continuous impervious surfaces data. *Landsc. Urban Plan.* 102 143–155. 10.1016/j.landurbplan.2011.03.017

[B71] Wang M. Markert B. Chen W. Peng C. Ouyang Z. (2012). Identification of heavy metal pollutants using multivariate analysis and effects of land uses on their accumulation in urban soils in Beijing, China. *Environ. Monit. Assess.* 184 5889–5897. 10.1007/s10661-011-2388-9 22068310

[B72] Wang Y. P. Shi J. Y. Wang H. Lin Q. Chen X. C. Chen Y. X. (2007). The influence of soil heavy metals pollution on soil microbial biomass, enzyme activity, and community composition near a copper smelter. *Ecotoxicol. Environ. Saf.* 67 75–81. 10.1016/j.ecoenv.2006.03.007 16828162

[B73] Wei B. G. Yang L. S. (2010). A review of heavy metal contaminations in urban soils, urban road dusts and agricultural soils from china. *Microchem. J.* 94 99–107. 10.1016/j.microc.2009.09.014

[B74] Wei Z. Wu S. Zhou S. Lin C. (2013). Installation of impervious surfaces in urban areas affects microbial biomass, activity (potential C mineralisation), and functional diversity of the fine earth. *Soil Res.* 51 59–67. 10.1071/SR12089

[B75] Wolch J. R. Byrne J. Newell J. P. (2014). Urban green space, public health, and environmental justice: the challenge of making cities ‘just green enough’. *Landsc. Urban Plan.* 125 234–244. 10.1016/j.landurbplan.2014.01.017

[B76] Xi C. Xia X. Ye Z. Ping Z. (2010). Heavy metal concentrations in roadside soils and correlation with urban traffic in Beijing, China. *J. Hazard. Mater.* 181 640–646. 10.1016/j.jhazmat.2010.05.060 20541319

[B77] Xie Y. Fan J. Zhu W. Amombo E. Lou Y. Chen L. (2016). Effect of heavy metals pollution on soil microbial diversity and bermudagrass genetic variation. *Front. Plant Sci.* 7:755. 10.3389/fpls.2016.00755 27303431 PMC4885870

[B78] Xu H. J. Li S. Su J. Q. Nie S. Gibson V. Li H. (2014). Does urbanization shape bacterial community composition in urban park soils? A case study in 16 representative Chinese cities based on the pyrosequencing method. *FEMS Microbiol. Ecol.* 87 182–192. 10.1111/1574-6941.12215 24117629

[B79] Xu X. Zhang Z. Hu S. Ruan Z. Jiang J. Chen C. (2017). Response of soil bacterial communities to lead and zinc pollution revealed by Illumina MiSeq sequencing investigation. *Environ. Sci. Pollut. Res.* 24 666–675. 10.1007/s11356-016-7826-3 27744590

[B80] Yan B. Li J. Xiao N. Qi Y. Fu G. Liu G. (2016). Urban-development- induced changes in the diversity and composition of the soil bacterial community in Beijing. *Sci. Rep.* 6:38811. 10.1038/srep38811 27934957 PMC5146926

[B81] Yan Y. Kuang W. Zhang C. Chen C. (2015). Impacts of impervious surfaces expansion on soil organic carbon–a spatially explicit study. *Sci. Rep.* 5:17905. 10.1038/srep17905 26642831 PMC4672273

[B82] Zeng J. Liu X. Song L. Lin X. Zhang H. Shen C. (2016). Nitrogen fertilization directly affects soil bacterial diversity and indirectly affects bacterial community composition. *Soil Biol. Biochem.* 92 41–49. 10.1016/j.soilbio.2015.09.018 22134642

[B83] Zhang X. Zhang X. Li G. (2013). Spatial variability of soil nutrients in urban ecological parks-A case study of the Beijing Olympic Forest Park. *Tsinghua Sci. Technol.* 53 90–95. 10.16511/j.cnki.qhdxxb.2013.01.015

[B84] Zhang X. Zhang X. Li G. (2015). The effect of texture and irrigation on the soil moisture vertical-temporal variability in an urban artificial landscape: a case study of Olympic Forest Park in Beijing. *Front. Environ. Sci. Eng.* 9 269–278. 10.1007/s11783-014-0672-y

[B85] Zhao D. Li F. Wang R. Yang Q. Ni H. (2012). Effect of soil sealing on the microbial biomass, N transformation and related enzyme activities at various depths of soils in urban area of Beijing, China. *J. Soils Sediments* 12 519–530. 10.1007/s11368-012-0472-6

[B86] Zhao D. Li F. Yang Q. Wang R. Song Y. Tao Y. (2013). The influence of different types of urban land use on soil microbial biomass and functional diversity in Beijing, China. *Soil Use Manage.* 29 230–239. 10.1111/sum.12034

