# Supplementary figures and images for: Impervious Surfaces Alter Soil Bacterial Communities in Urban Areas: A Case Study in Beijing, China
Source: Front Microbiol. 2018 Feb 27;9:226. doi: 10.3389/fmicb.2018.00226 (PMC5839015; doi:10.3389/fmicb.2018.00226)

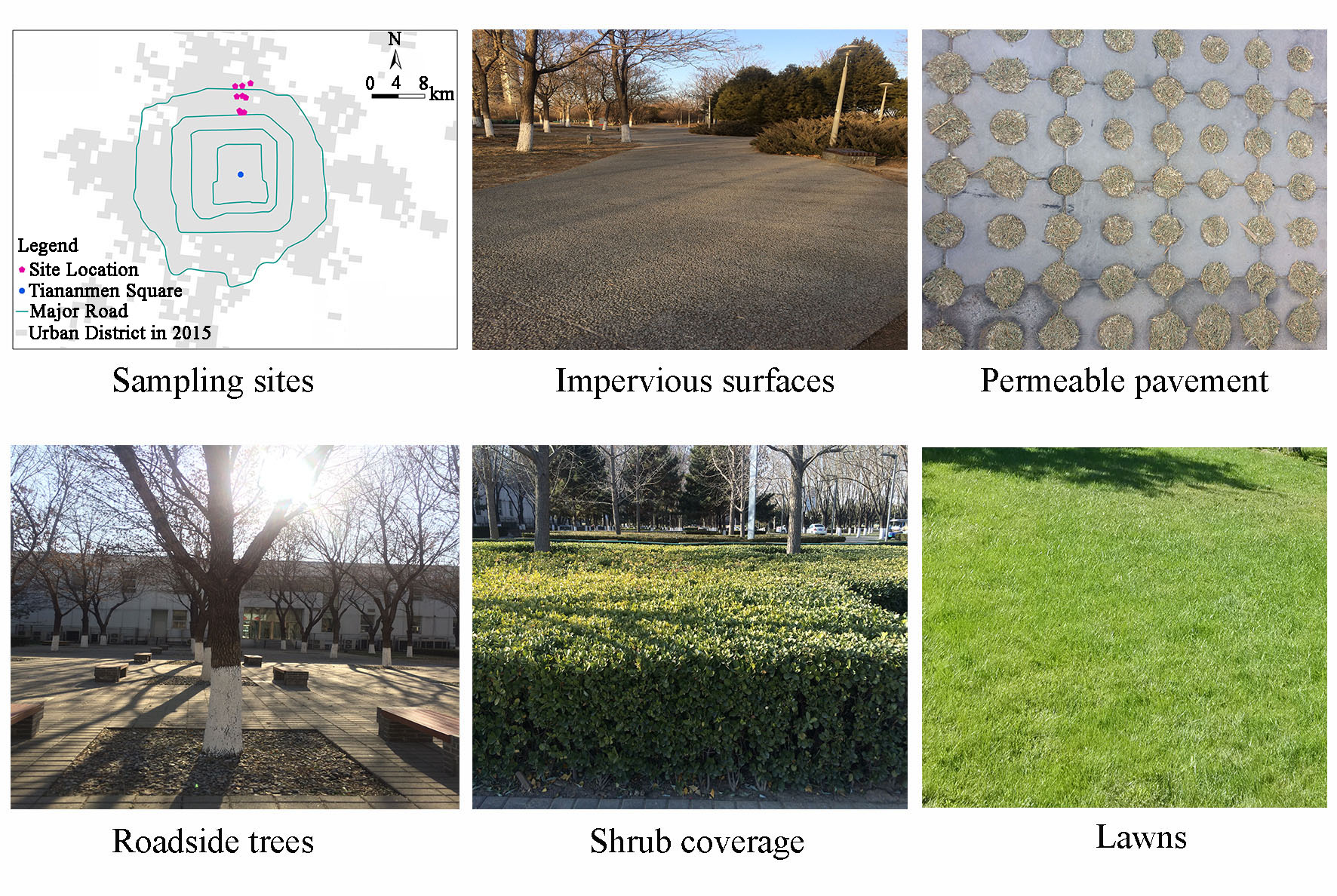

Supplement: Supplementary file 1 [file Image_1.JPEG]

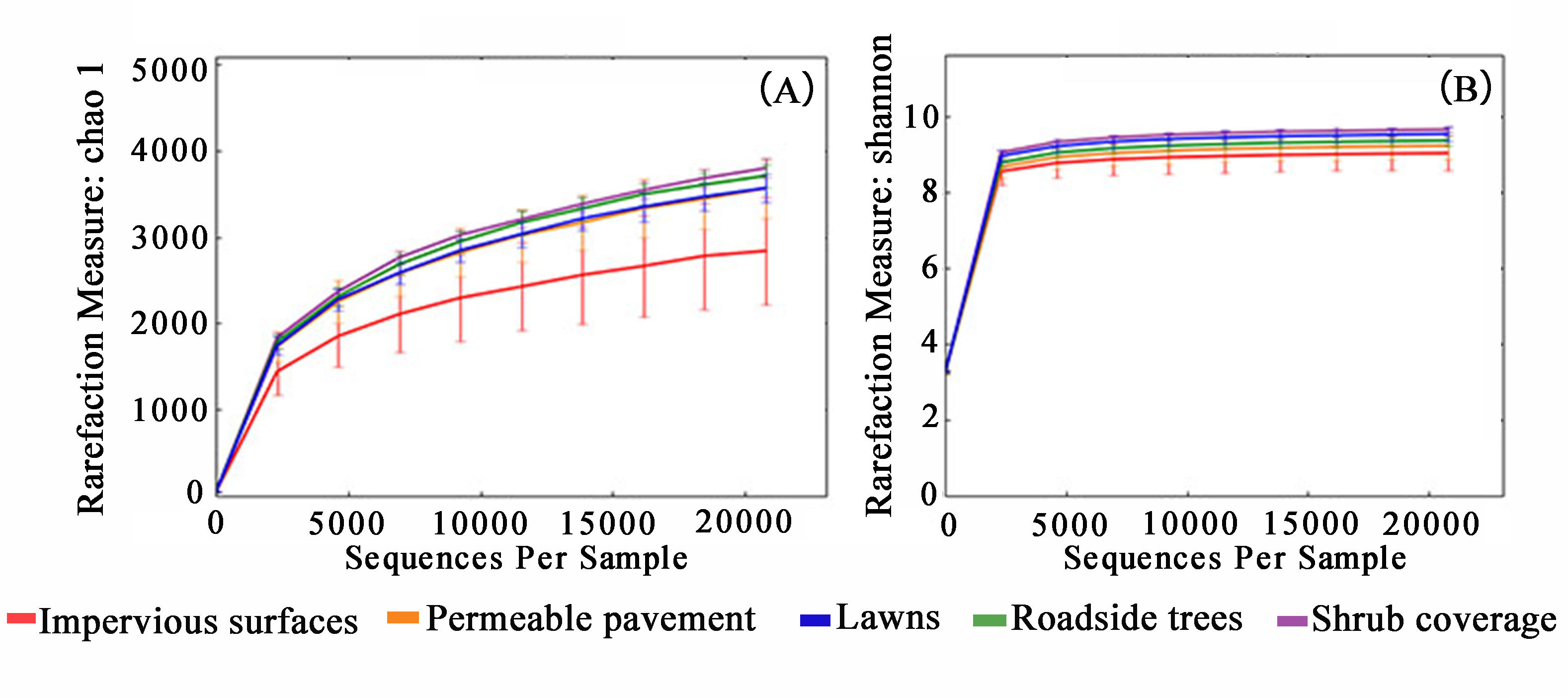

Supplement: Supplementary file 3 [file Image_3.JPEG]
