# Supplementary material for: Impervious Surfaces Alter Soil Bacterial Communities in Urban Areas: A Case Study in Beijing, China
Source: Front Microbiol. 2018 Feb 27;9:226. doi: 10.3389/fmicb.2018.00226 (PMC5839015; doi:10.3389/fmicb.2018.00226)
Supplement: Supplementary file 5 [file Data_Sheet_1.DOCX]

Table S1: Number of sequence reads (before (BF) and after (AF) quality filtering using the Quantitative Insights into Microbial Ecology (QIIME) software package) and OTUs classified from after quality filtering sequences. Different letters indicate different land cover types. I: Impervious surfaces; L: Lawns; P: Permeable pavement; R: Roadside trees; S: Shrub coverage.

| Sample ID | BF Sequences | AF Sequences | OTUs |
| --- | --- | --- | --- |
| I1 | 80369 | 58515 | 2838 |
| I2 | 75404 | 53554 | 2466 |
| I3 | 80578 | 55912 | 2196 |
| I4 | 75915 | 49754 | 1840 |
| I5 | 73161 | 53008 | 2308 |
| I6 | 58819 | 36189 | 2334 |
| I7 | 70893 | 46553 | 3342 |
| I8 | 58236 | 36499 | 3113 |
| I9 | 83890 | 51638 | 3542 |
| L1 | 42082 | 25999 | 2639 |
| L2 | 45439 | 30515 | 3033 |
| L3 | 65205 | 44239 | 3368 |
| L4 | 41341 | 24570 | 2685 |
| L5 | 43789 | 28523 | 2643 |
| L6 | 64913 | 41773 | 2971 |
| L7 | 49249 | 34338 | 3067 |
| L8 | 59181 | 34695 | 2854 |
| L9 | 75620 | 44935 | 3480 |
| P1 | 95757 | 58928 | 3829 |
| P2 | 99122 | 62506 | 3743 |
| P3 | 71469 | 44459 | 3589 |
| P4 | 82157 | 55755 | 2911 |
| P5 | 56401 | 32316 | 2775 |
| P6 | 72822 | 41766 | 2577 |
| P7 | 87812 | 54624 | 3495 |
| P8 | 71397 | 45190 | 3533 |
| P9 | 84353 | 51554 | 3280 |
| R1 | 82502 | 50750 | 3529 |
| R2 | 76018 | 56671 | 3624 |
| R3 | 68907 | 44711 | 3599 |
| R4 | 67680 | 45485 | 3234 |
| R5 | 67637 | 46971 | 3203 |
| R6 | 60870 | 38592 | 3052 |
| R7 | 66425 | 44369 | 3356 |
| R8 | 59953 | 38325 | 3291 |
| R9 | 85784 | 54847 | 3621 |
| S1 | 71192 | 47274 | 3540 |
| S2 | 59704 | 41001 | 3284 |
| S3 | 74058 | 48175 | 3472 |
| S4 | 84568 | 55567 | 3639 |
| S5 | 74932 | 49297 | 3709 |
| S6 | 41181 | 23065 | 2680 |
| S7 | 70243 | 43980 | 3429 |
| S8 | 58136 | 34130 | 3213 |
| S9 | 42354 | 25608 | 2780 |

Table S2: Relative abundance of the main bacterial phyla classified with RDP taxonomy in five land cover types (values represent % of total non-redundant sequences). Different letters indicate statistical significance at *P* < 0.05 among land cover types.

| Phylum | Impervious surfaces | Permeable pavement | Shrub coverage | Lawns | Roadside trees |
| --- | --- | --- | --- | --- | --- |
| *Proteobacteria* | 27.5b | 35.0a | 40.9a | 38.9a | 39.02a |
| *Actinobacteria* | 16.1c | 23.9a | 16.1bc | 13.3c | 19.1b |
| *Acidobacteria* | 15.0a | 11.5b | 15.6a | 16.0a | 16.5a |
| *Chloroflexi* | 14.4a | 9.6b | 7.4b | 8.5b | 8.2b |
| *Bacteroidetes* | 3.8b | 4.9a | 6.7a | 7.7a | 4.5b |
| *Firmicutes* | 5.4a | 2.4b | 0.8c | 2.0b | 2.3b |
| others | 17.9a | 12.7b | 12.4b | 13.7b | 10.5c |

Table S3: Relative abundances of the dominant bacterial classes (a), orders (b), families (c), genera (d) and OTUs (e) across land cover types, and only those with relative abundance ≥ 0.5% at least at one sampling time were shown.

| Land cover types | Impervious surfaces | Lawns | Permeable pavement | Roadside trees | Shrub coverage |
| --- | --- | --- | --- | --- | --- |
| **(a) Dominant Classes (35)** | | |  |  |  |
| *Alphaproteobacteria* | 8.3 | 15.9 | 22.2 | 19.7 | 16.9 |
| *Acidobacteria* | 13.3 | 15.8 | 8.1 | 14.9 | 13.8 |
| *Actinobacteria* | 1.3 | 5.2 | 16.7 | 9.0 | 5.5 |
| *Betaproteobacteria* | 6.1 | 7.1 | 6.4 | 7.1 | 7.5 |
| *Gammaproteobacteria* | 2.8 | 6.9 | 3.4 | 6.5 | 8.3 |
| *Deltaproteobacteria* | 8.4 | 7.4 | 4.7 | 5.3 | 7.6 |
| *Thermoleophilia* | 5.1 | 2.8 | 3.1 | 4.3 | 2.8 |
| *Gemmatimonadetes* | 5.9 | 2.9 | 2.0 | 3.6 | 3.2 |
| *Acidimicrobiia* | 2.4 | 3.2 | 3.2 | 3.3 | 5.7 |
| *KD4-96* | 3.1 | 1.5 | 1.8 | 2.9 | 1.7 |
| *Sphingobacteriia* | 0.3 | 4.1 | 3.0 | 2.1 | 3.0 |
| *Cytophagia* | 0.2 | 3.7 | 2.9 | 1.9 | 3.2 |
| *Holophagae* | 1.9 | 1.7 | 2.1 | 1.8 | 1.9 |
| *Anaerolineae* | 5.7 | 3.0 | 1.8 | 1.5 | 1.8 |
| *MB-A2-108* | 4.1 | 0.9 | 0.8 | 1.4 | 1.0 |
| *OPB35_soil_group* | 0.3 | 1.7 | 1.1 | 1.1 | 1.5 |
| *Nitrospira* | 4.5 | 1.2 | 0.5 | 1.0 | 1.2 |
| *Bacilli* | 4.7 | 1.1 | 1.7 | 0.9 | 0.7 |
| *Thermomicrobia* | 0.3 | 0.3 | 1.3 | 0.9 | 0.7 |
| *Spartobacteria* | 0.0 | 0.8 | 0.6 | 0.8 | 0.4 |
| *Gitt-GS-136* | 1.7 | 0.5 | 0.7 | 0.7 | 0.6 |
| *OM190* | 0.2 | 1.2 | 0.2 | 0.7 | 1.2 |
| *Chloroflexia* | 0.2 | 0.7 | 1.1 | 0.6 | 0.3 |
| *Phycisphaerae* | 0.2 | 0.8 | 0.4 | 0.5 | 0.8 |
| *TK10* | 0.4 | 0.4 | 0.5 | 0.4 | 0.7 |
| *TakashiAC-B11* | 0.0 | 0.1 | 0.9 | 0.3 | 0.3 |
| *Flavobacteriia* | 0.0 | 0.4 | 0.8 | 0.3 | 0.5 |
| *Ardenticatenia* | 0.6 | 0.7 | 0.4 | 0.3 | 0.5 |
| *S085* | 1.0 | 0.5 | 0.3 | 0.3 | 0.4 |
| *Subgroup_22* | 0.9 | 0.6 | 0.0 | 0.3 | 0.5 |
| *JG30-KF-CM66* | 0.6 | 0.2 | 0.2 | 0.1 | 0.2 |
| *Clostridia* | 1.3 | 0.1 | 0.1 | 0.1 | 0.1 |
| *Bacteroidia* | 1.0 | 0.1 | 0.1 | 0.1 | 0.0 |
| *Thermotogae* | 2.8 | 0.0 | 0.0 | 0.0 | 0.0 |
| *Cyanobacteria* | 0.0 | 0.0 | 0.9 | 0.0 | 0.0 |
| unidentified | 6.7 | 3.1 | 3.0 | 2.8 | 2.6 |
| **(b) Dominant Orders (35)** | | |  |  |  |
| *Rhizobiales* | 5.4 | 8.3 | 8.4 | 10.8 | 9.3 |
| *Subgroup_6* | 6.3 | 8.5 | 4.8 | 8.3 | 9.1 |
| *Rhodospirillales* | 2.0 | 4.4 | 6.0 | 5.2 | 4.1 |
| *Xanthomonadales* | 1.0 | 5.3 | 2.2 | 4.6 | 6.2 |
| *Subgroup_4* | 1.0 | 3.5 | 2.1 | 4.3 | 2.3 |
| *Acidimicrobiales* | 2.4 | 3.2 | 3.2 | 3.3 | 5.7 |
| *Micrococcales* | 0.7 | 2.6 | 8.2 | 3.2 | 2.2 |
| *Nitrosomonadales* | 3.7 | 3.8 | 1.4 | 3.1 | 3.2 |
| *Gemmatimonadales* | 5.3 | 2.2 | 1.3 | 3.0 | 2.4 |
| *Myxococcales* | 1.3 | 3.3 | 3.2 | 2.8 | 4.1 |
| *Sphingomonadales* | 0.4 | 2.3 | 5.0 | 2.7 | 2.0 |
| *Solirubrobacterales* | 0.9 | 1.1 | 2.1 | 2.2 | 1.3 |
| *Sphingobacteriales* | 0.3 | 4.1 | 3.0 | 2.1 | 3.0 |
| *Gaiellales* | 4.2 | 1.7 | 1.0 | 2.1 | 1.5 |
| *Cytophagales* | 0.2 | 3.7 | 2.9 | 1.9 | 3.2 |
| *Burkholderiales* | 0.9 | 1.6 | 3.8 | 1.6 | 1.6 |
| *Anaerolineales* | 5.7 | 3.0 | 1.8 | 1.5 | 1.8 |
| *Propionibacteriales* | 0.2 | 0.8 | 2.8 | 1.5 | 0.9 |
| *TRA3-20* | 0.9 | 1.1 | 0.8 | 1.3 | 2.0 |
| *Pseudomonadales* | 0.3 | 0.8 | 0.3 | 1.2 | 1.0 |
| *GR-WP33-30* | 3.7 | 1.6 | 0.5 | 1.2 | 1.4 |
| *Subgroup_10* | 0.5 | 1.1 | 1.3 | 1.1 | 1.4 |
| *Nitrospirales* | 4.5 | 1.2 | 0.5 | 1.0 | 1.2 |
| *Micromonosporales* | 0.1 | 0.4 | 1.2 | 1.0 | 0.5 |
| *Bacillales* | 4.4 | 1.1 | 1.6 | 0.9 | 0.7 |
| *JG30-KF-CM45* | 0.3 | 0.3 | 1.0 | 0.7 | 0.6 |
| *Subgroup_7* | 1.4 | 0.7 | 0.7 | 0.7 | 0.6 |
| *Subgroup_3* | 0.8 | 1.0 | 0.6 | 0.7 | 0.8 |
| *Corynebacteriales* | 0.0 | 0.2 | 0.7 | 0.6 | 0.8 |
| *Desulfobacterales* | 0.8 | 1.0 | 0.3 | 0.6 | 0.7 |
| *Subgroup_17* | 0.8 | 0.7 | 0.1 | 0.5 | 0.6 |
| *Caulobacterales* | 0.1 | 0.5 | 0.9 | 0.4 | 0.9 |
| *Phycisphaerales* | 0.1 | 0.7 | 0.3 | 0.4 | 0.7 |
| *Sh765B-TzT-29* | 0.6 | 0.5 | 0.0 | 0.1 | 0.9 |
| unidentified | 21.3 | 12.5 | 10.6 | 12.0 | 12.2 |
| **(c) Dominant Families (39)** | | |  |  |  |
| *Xanthobacteraceae* | 1.1 | 1.5 | 0.9 | 1.9 | 2.0 |
| *0319-6A21* | 2.6 | 0.5 | 0.2 | 0.5 | 0.5 |
| *Rhodobiaceae* | 0.7 | 0.7 | 0.2 | 0.4 | 0.6 |
| *Comamonadaceae* | 0.8 | 1.0 | 1.3 | 1.0 | 1.1 |
| *MNH2* | 0.5 | 0.5 | 0.1 | 0.3 | 0.3 |
| *Micrococcaceae* | 0.7 | 2.0 | 6.7 | 2.3 | 1.4 |
| *Nitrosomonadaceae* | 3.5 | 3.8 | 1.4 | 3.1 | 3.2 |
| *Rhodospirillaceae* | 0.5 | 2.3 | 4.1 | 3.2 | 1.7 |
| *RB41* | 0.8 | 2.4 | 1.3 | 3.5 | 1.4 |
| *OM1_clade* | 1.3 | 1.3 | 0.5 | 1.0 | 2.5 |
| *Ectothiorhodospiraceae* | 0.9 | 0.1 | 0.0 | 0.1 | 0.1 |
| *Paenibacillaceae* | 0.8 | 0.1 | 0.1 | 0.1 | 0.1 |
| *Gaiellaceae* | 2.2 | 0.8 | 0.5 | 1.2 | 0.9 |
| *Anaerolineaceae* | 5.7 | 3.0 | 1.8 | 1.5 | 1.8 |
| *UnknownFamily* | 0.6 | 1.1 | 1.1 | 1.0 | 1.0 |
| *Nitrospinaceae* | 0.7 | 1.0 | 0.3 | 0.5 | 0.7 |
| *unidentified* | 45.9 | 35.0 | 25.4 | 33.2 | 36.9 |
| *Bacteroidaceae* | 0.5 | 0.0 | 0.1 | 0.1 | 0.0 |
| *Nitrospiraceae* | 1.6 | 0.7 | 0.3 | 0.6 | 0.7 |
| *Gemmatimonadaceae* | 5.3 | 2.2 | 1.3 | 3.0 | 2.4 |
| *Bacillaceae* | 3.3 | 0.9 | 0.7 | 0.7 | 0.6 |
| *Methylobacteriaceae* | 0.6 | 0.8 | 1.9 | 1.0 | 0.7 |
| *Hyphomicrobiaceae* | 0.9 | 1.4 | 1.7 | 1.6 | 1.6 |
| *Thermotogaceae* | 2.8 | 0.0 | 0.0 | 0.0 | 0.0 |
| *Acidimicrobiaceae* | 0.2 | 0.7 | 0.7 | 0.7 | 0.9 |
| *Saprospiraceae* | 0.1 | 0.8 | 0.3 | 0.3 | 0.4 |
| *Haliangiaceae* | 0.4 | 1.2 | 0.6 | 0.9 | 0.9 |
| *Nocardioidaceae* | 0.1 | 0.7 | 2.8 | 1.4 | 0.9 |
| *Roseiflexaceae* | 0.2 | 0.6 | 0.5 | 0.5 | 0.2 |
| *Cytophagaceae* | 0.2 | 3.7 | 2.8 | 1.9 | 3.2 |
| *Pseudomonadaceae* | 0.3 | 0.7 | 0.2 | 1.2 | 0.9 |
| *Chitinophagaceae* | 0.1 | 2.5 | 1.9 | 1.5 | 2.0 |
| *ABS-19* | 0.3 | 0.8 | 1.3 | 1.0 | 1.0 |
| *Xanthomonadaceae* | 0.2 | 1.3 | 0.8 | 0.9 | 0.8 |
| *Sphingomonadaceae* | 0.4 | 1.9 | 4.5 | 2.5 | 1.8 |
| *BIrii41* | 0.1 | 0.8 | 0.3 | 0.4 | 0.8 |
| *Xanthomonadales_Incertae_Sedis* | 0.2 | 1.8 | 1.0 | 2.2 | 2.8 |
| *Bradyrhizobiaceae* | 0.1 | 0.6 | 0.8 | 1.1 | 0.7 |
| *Phycisphaeraceae* | 0.1 | 0.7 | 0.3 | 0.4 | 0.7 |
| *Rhizobiaceae* | 0.1 | 0.4 | 0.7 | 1.2 | 0.4 |
| *Oxalobacteraceae* | 0.0 | 0.1 | 2.3 | 0.2 | 0.1 |
| *Caulobacteraceae* | 0.0 | 0.1 | 0.7 | 0.2 | 0.3 |
| *Rhodobacteraceae* | 0.2 | 0.2 | 1.7 | 0.5 | 0.4 |
| *Streptomycetaceae* | 0.1 | 0.4 | 1.0 | 1.0 | 0.5 |
| *480-2* | 0.4 | 0.4 | 0.6 | 0.8 | 0.5 |
| *Flavobacteriaceae* | 0.0 | 0.3 | 0.7 | 0.3 | 0.4 |
| *Sphingobacteriaceae* | 0.0 | 0.1 | 0.6 | 0.1 | 0.1 |
| *Pseudonocardiaceae* | 0.0 | 0.2 | 0.8 | 0.7 | 0.1 |
| *Micromonosporaceae* | 0.1 | 0.4 | 1.2 | 1.0 | 0.5 |
| *Geodermatophilaceae* | 0.0 | 0.3 | 1.2 | 0.5 | 0.3 |
| *Planococcaceae* | 0.1 | 0.0 | 0.5 | 0.0 | 0.0 |
| *Intrasporangiaceae* | 0.0 | 0.2 | 0.6 | 0.3 | 0.2 |
| *FamilyI* | 0.0 | 0.0 | 0.8 | 0.0 | 0.0 |
| *RhodospirillalesIncertaeSedis* | 0.0 | 0.4 | 0.6 | 0.5 | 0.5 |
| *Hyphomonadaceae* | 0.1 | 0.5 | 0.1 | 0.2 | 0.6 |
| *RhizobialesIncertaeSedis* | 0.4 | 0.4 | 0.4 | 0.6 | 0.8 |
| *Mycobacteriaceae* | 0.0 | 0.2 | 0.4 | 0.5 | 0.6 |
| *Sandaracinaceae* | 0.1 | 0.2 | 0.4 | 0.5 | 0.9 |
| **(d) Dominant Genera (35)** | 0.0 | 0.0 | 0.0 | 0.0 | 0.0 |
| *Acidibacter* | 0.0 | 0.6 | 0.2 | 0.3 | 0.7 |
| *Acidiferrobacter* | 0.9 | 0.1 | 0.0 | 0.1 | 0.1 |
| *Adhaeribacter* | 0.0 | 0.4 | 0.5 | 0.1 | 0.2 |
| *Arthrobacter* | 0.7 | 2.0 | 6.0 | 2.2 | 1.4 |
| *Bacillus* | 2.9 | 0.9 | 0.6 | 0.7 | 0.5 |
| *Bacteroides* | 0.5 | 0.0 | 0.1 | 0.1 | 0.0 |
| *Blastococcus* | 0.0 | 0.3 | 1.0 | 0.4 | 0.2 |
| *Bradyrhizobium* | 0.1 | 0.5 | 0.4 | 1.0 | 0.6 |
| *CandidatusEntotheonella* | 0.3 | 0.7 | 0.2 | 0.4 | 0.5 |
| *Chryseolinea* | 0.0 | 2.1 | 0.4 | 0.8 | 1.6 |
| *Devosia* | 0.1 | 0.3 | 0.9 | 0.3 | 0.3 |
| *Flavobacterium* | 0.0 | 0.3 | 0.7 | 0.3 | 0.4 |
| *Gaiella* | 2.2 | 0.8 | 0.5 | 1.2 | 0.9 |
| *GAL15* | 2.8 | 0.0 | 0.0 | 0.0 | 0.0 |
| *Haliangium* | 0.4 | 1.2 | 0.6 | 0.9 | 0.9 |
| *Kocuria* | 0.0 | 0.0 | 0.7 | 0.1 | 0.0 |
| *Lysobacter* | 0.1 | 0.9 | 0.2 | 0.6 | 0.4 |
| *Marmoricola* | 0.1 | 0.3 | 1.0 | 0.5 | 0.3 |
| *Massilia* | 0.0 | 0.0 | 1.5 | 0.1 | 0.0 |
| *Microvirga* | 0.0 | 0.6 | 1.7 | 0.9 | 0.5 |
| *Mycobacterium* | 0.0 | 0.2 | 0.4 | 0.5 | 0.6 |
| *Nitrospira* | 1.4 | 0.6 | 0.3 | 0.6 | 0.7 |
| *Nocardioides* | 0.0 | 0.3 | 1.6 | 0.7 | 0.5 |
| *Nordella* | 0.4 | 0.3 | 0.3 | 0.5 | 0.4 |
| *Novosphingobium* | 0.1 | 0.0 | 0.5 | 0.4 | 0.2 |
| *Paracocccus* | 0.0 | 0.0 | 0.6 | 0.1 | 0.0 |
| *Pedomicrobium* | 0.2 | 0.5 | 0.4 | 0.5 | 0.7 |
| *Pseudomonas* | 0.3 | 0.7 | 0.2 | 1.2 | 0.9 |
| *Rhizobium* | 0.1 | 0.3 | 0.6 | 1.2 | 0.4 |
| *Roseiflexus* | 0.2 | 0.6 | 0.5 | 0.5 | 0.2 |
| *Rubellimicrobium* | 0.0 | 0.0 | 0.8 | 0.1 | 0.0 |
| *Skermanella* | 0.0 | 0.7 | 3.2 | 1.4 | 0.6 |
| *Sphingomonas* | 0.3 | 1.9 | 3.9 | 2.0 | 1.6 |
| *Steroidobacter* | 0.1 | 0.9 | 0.7 | 1.5 | 1.4 |
| *Streptomyces* | 0.1 | 0.4 | 1.0 | 1.0 | 0.5 |
| unidentified | 76.0 | 67.3 | 47.0 | 62.0 | 67.2 |
| **(e) Dominant OTUs (74)** | | |  |  |  |
| OTU12（*GAL15*） | 1.4 | 0.0 | 0.0 | 0.0 | 0.0 |
| OTU27（*Gaiella*） | 1.0 | 0.2 | 0.1 | 0.2 | 0.2 |
| OTU83（*Bacillus*） | 0.8 | 0.2 | 0.2 | 0.3 | 0.2 |
| OTU6（*Bacillus*） | 0.8 | 0.6 | 0.1 | 0.2 | 0.2 |
| OTU177（*Actinobacteria*） | 0.7 | 0.3 | 0.2 | 0.5 | 0.4 |
| OTU1 (*Arthrobacter*) | 0.6 | 2.0 | 5.7 | 2.2 | 1.4 |
| OTU746 (*GAL15*) | 0.6 | 0.0 | 0.0 | 0.0 | 0.0 |
| OTU32 (*GAL15*) | 0.5 | 0.0 | 0.0 | 0.0 | 0.0 |
| OTU29 (*Bacillus*) | 0.5 | 0.0 | 0.1 | 0.1 | 0.1 |
| OTU37 (*Nitrospira*) | 0.4 | 0.6 | 0.2 | 0.5 | 0.5 |
| OTU48 (*Variibacter*) | 0.3 | 0.4 | 0.3 | 0.7 | 0.7 |
| OTU92 (*Sphingomonas*) | 0.1 | 0.4 | 1.7 | 0.7 | 0.4 |
| OTU54 (*Pseudomonas*) | 0.1 | 0.2 | 0.1 | 0.9 | 0.4 |
| OTU62 (*Marmoricola*) | 0.1 | 0.3 | 0.9 | 0.4 | 0.3 |
| OTU18 (*Sphingomonas*) | 0.1 | 1.3 | 1.4 | 1.0 | 1.0 |
| OTU3 (*Bradyrhizobium*) | 0.1 | 0.5 | 0.3 | 1.0 | 0.5 |
| OTU36 (*Steroidobacter*) | 0.1 | 0.8 | 0.6 | 1.1 | 1.3 |
| OTU28 (*Rhizobium*) | 0.1 | 0.3 | 0.1 | 1.1 | 0.3 |
| OTU65 (*Streptomyces*) | 0.1 | 0.2 | 0.6 | 0.7 | 0.2 |
| OTU187 (*Novosphingobium*) | 0.1 | 0.0 | 0.5 | 0.4 | 0.2 |
| OTU50 (*Blastococcus*) | 0.0 | 0.3 | 1.0 | 0.4 | 0.2 |
| OTU51 (*Mycobacterium*) | 0.0 | 0.2 | 0.3 | 0.4 | 0.5 |
| OTU7 (*Microvirga*) | 0.0 | 0.5 | 1.6 | 0.9 | 0.4 |
| OTU8817 (*Skermanella*) | 0.0 | 0.3 | 0.9 | 0.5 | 0.2 |
| OTU2 (*Skermanella*) | 0.0 | 0.3 | 2.2 | 0.8 | 0.4 |
| OTU773 (*Sphingomonas*) | 0.0 | 0.1 | 0.6 | 0.2 | 0.1 |
| OTU256 (*Massilia*) | 0.0 | 0.0 | 1.1 | 0.1 | 0.0 |
| OTU98 (*Rubellimicrobium*) | 0.0 | 0.0 | 0.7 | 0.1 | 0.0 |
| OTU17 (*Chryseolinea*) | 0.0 | 1.2 | 0.1 | 0.5 | 1.1 |
| OTU49 (*Paracocccus*) | 0.0 | 0.0 | 0.6 | 0.1 | 0.0 |
| OTU93 (*Acidibacter*) | 0.0 | 0.5 | 0.2 | 0.2 | 0.7 |
| OTU25 (*Kocuria*) | 0.0 | 0.0 | 0.7 | 0.1 | 0.0 |
| OTU42 (*Chryseolinea*) | 0.0 | 0.6 | 0.3 | 0.3 | 0.5 |
| OTU43 (*Nocardioides*) | 0.0 | 0.0 | 0.6 | 0.1 | 0.1 |
| OTU11（unidentified） | 1.6 | 1.0 | 1.1 | 1.9 | 1.0 |
| OTU14（unidentified） | 1.4 | 0.3 | 0.3 | 0.6 | 0.3 |
| OTU9（unidentified） | 1.1 | 0.1 | 0.1 | 0.2 | 0.1 |
| OTU23（unidentified） | 1.0 | 0.3 | 0.0 | 0.2 | 0.2 |
| OTU15（unidentified） | 0.9 | 0.0 | 0.1 | 0.1 | 0.1 |
| OTU13（unidentified） | 0.8 | 0.0 | 0.0 | 0.0 | 0.0 |
| OTU8（unidentified） | 0.7 | 0.8 | 0.3 | 0.8 | 0.9 |
| OTU20（unidentified） | 0.7 | 0.3 | 0.2 | 0.5 | 0.3 |
| OTU19（unidentified） | 0.7 | 0.7 | 0.2 | 0.4 | 0.6 |
| OTU105（unidentified） | 0.7 | 0.1 | 0.0 | 0.2 | 0.1 |
| OTU119（unidentified） | 0.7 | 0.2 | 0.1 | 0.1 | 0.1 |
| OTU128（unidentified） | 0.6 | 0.4 | 0.1 | 0.4 | 0.4 |
| OTU55（unidentified） | 0.6 | 0.2 | 0.0 | 0.1 | 0.1 |
| OTU22（unidentified） | 0.6 | 0.1 | 0.0 | 0.1 | 0.1 |
| OTU26（unidentified） | 0.6 | 1.0 | 0.3 | 0.9 | 0.7 |
| OTU120（unidentified） | 0.6 | 0.0 | 0.0 | 0.0 | 0.0 |
| OTU40（unidentified） | 0.6 | 0.3 | 0.2 | 0.3 | 0.2 |
| OTU39（unidentified） | 0.5 | 0.1 | 0.1 | 0.1 | 0.0 |
| OTU75（unidentified） | 0.5 | 0.0 | 0.0 | 0.0 | 0.0 |
| OTU24（unidentified） | 0.5 | 0.5 | 0.1 | 0.3 | 0.3 |
| OTU30（unidentified） | 0.5 | 0.0 | 0.0 | 0.0 | 0.0 |
| OTU53（unidentified） | 0.5 | 0.8 | 0.7 | 0.9 | 1.0 |
| OTU84（unidentified） | 0.4 | 0.5 | 0.1 | 0.5 | 0.5 |
| OTU45（unidentified） | 0.4 | 0.5 | 0.1 | 0.3 | 0.7 |
| OTU180（unidentified） | 0.3 | 0.4 | 0.2 | 0.6 | 0.5 |
| OTU106（unidentified） | 0.3 | 0.3 | 0.1 | 0.3 | 0.6 |
| OTU31（unidentified） | 0.3 | 0.7 | 0.1 | 0.7 | 1.2 |
| OTU94（unidentified） | 0.2 | 1.7 | 0.7 | 2.1 | 1.0 |
| OTU46（unidentified） | 0.2 | 0.5 | 0.0 | 0.2 | 0.4 |
| OTU35（unidentified） | 0.1 | 0.3 | 0.0 | 0.1 | 1.1 |
| OTU86（unidentified） | 0.1 | 0.1 | 0.6 | 0.4 | 0.2 |
| OTU64（unidentified） | 0.1 | 0.6 | 0.1 | 0.3 | 0.7 |
| OTU4（unidentified） | 0.1 | 1.2 | 0.4 | 1.6 | 0.8 |
| OTU299（unidentified） | 0.1 | 0.6 | 0.2 | 0.4 | 0.6 |
| OTU71（unidentified） | 0.0 | 0.4 | 0.3 | 1.2 | 0.8 |
| OTU171（unidentified） | 0.0 | 0.2 | 0.6 | 0.3 | 0.2 |
| OTU63（unidentified） | 0.0 | 0.8 | 0.1 | 0.2 | 0.7 |
| OTU213（unidentified） | 0.0 | 0.2 | 0.6 | 0.3 | 0.2 |
| OTU148（unidentified） | 0.0 | 0.5 | 0.0 | 0.1 | 0.2 |
| OTU198 (unidentified） | 0.0 | 0.0 | 0.5 | 0.0 | 0.0 |
